# A Chimeric HIV-1 gp120 Fused with Vaccinia Virus 14K (A27) Protein as an HIV Immunogen

**DOI:** 10.1371/journal.pone.0133595

**Published:** 2015-07-24

**Authors:** Aneesh Vijayan, Juan García-Arriaza, Suresh C. Raman, José Javier Conesa, Francisco Javier Chichón, César Santiago, Carlos Óscar S. Sorzano, José L. Carrascosa, Mariano Esteban

**Affiliations:** 1 Department of Molecular and Cellular Biology, Centro Nacional de Biotecnología, Consejo Superior de Investigaciones Científicas (CNB-CSIC), Madrid, Spain; 2 Department of Structure of Macromolecules, Centro Nacional de Biotecnología, Consejo Superior de Investigaciones Científicas (CNB-CSIC), Madrid, Spain; 3 X-ray Crystallization Unit, Centro Nacional de Biotecnología, Consejo Superior de Investigaciones Científicas (CNB-CSIC), Madrid, Spain; 4 Biocomputing Unit, Centro Nacional de Biotecnología, Consejo Superior de Investigaciones Científicas (CNB-CSIC), Madrid, Spain; University of Massachusetts Medical Center, UNITED STATES

## Abstract

In the HIV vaccine field, there is a need to produce highly immunogenic forms of the Env protein with the capacity to trigger broad B and T-cell responses. Here, we report the generation and characterization of a chimeric HIV-1 gp120 protein (termed gp120-14K) by fusing gp120 from clade B with the vaccinia virus (VACV) 14K oligomeric protein (derived from A27L gene). Stable CHO cell lines expressing HIV-1 gp120-14K protein were generated and the protein purified was characterized by size exclusion chromatography, electron microscopy and binding to anti-Env antibodies. These approaches indicate that gp120-14K protein is oligomeric and reacts with a wide spectrum of HIV-1 neutralizing antibodies. Furthermore, in human monocyte-derived dendritic cells (moDCs), gp120-14K protein upregulates the levels of several proinflammatory cytokines and chemokines associated with Th1 innate immune responses (IL-1β, IFN-γ, IL-6, IL-8, IL-12, RANTES). Moreover, we showed in a murine model, that a heterologous prime/boost immunization protocol consisting of a DNA prime with a plasmid expressing gp120-14K protein followed by a boost with MVA-B [a recombinant modified vaccinia virus Ankara (MVA) expressing HIV-1 gp120, Gag, Pol and Nef antigens from clade B], generates stronger, more polyfunctional, and greater effector memory HIV-1-specific CD4^+^ and CD8^+^ T-cell immune responses, than immunization with DNA-gp120/MVA-B. The DNA/MVA protocol was superior to immunization with the combination of protein/MVA and the latter was superior to a prime/boost of MVA/MVA or protein/protein. In addition, these immunization protocols enhanced antibody responses against gp120 of the class IgG2a and IgG3, together favoring a Th1 humoral immune response. These results demonstrate that fusing HIV-1 gp120 with VACV 14K forms an oligomeric protein which is highly antigenic as it activates a Th1 innate immune response in human moDCs, and in vaccinated mice triggers polyfunctional HIV-1-specific adaptive and memory T-cell immune responses, as well as humoral responses. This novel HIV-1 gp120-14K immunogen might be considered as an HIV vaccine candidate for broad T and B-cell immune responses.

## Introduction

Acquired Immunodeficiency Syndrome (AIDS) is a scourge on mankind with an estimated 39 million deaths so far since the discovery of HIV-1, and over 35 million cases reported in 2013 (WHO Report October, 2014). Emergence of drug resistant strains and the high mutation rate of HIV-1 are the main obstacles in developing an effective vaccine against HIV/AIDS [1, 2]. Among the different HIV/AIDS vaccine candidates developed, the HIV-1 envelope glycoprotein stands out to be the most promising one [3, 4]. The precursor HIV-1 envelope protein exists as a polyprotein, known as gp160, which subsequently is cleaved into the receptor binding domain (gp120) and the membrane binding domain (gp41) [5]. The HIV-1 gp120 protein adopts conformational changes upon binding to the cell surface receptor CD4 and co-receptors CCR5 and CXCR4, thereby assisting viral entry into the cells and is therefore an attractive target for the immune system [6–8]. A small cohort of infected individuals (10–25%) is able to generate broadly neutralizing antibodies (bnAbs), suggesting that a viable gp120-based vaccine against HIV/AIDS is feasible [9, 10]. Generating an Env protein which mimics the native conformation is a long sought goal in HIV/AIDS vaccine development since the use of monomeric gp120 in clinical trials ended in failures with the exception of RV144 phase III clinical trial that showed a modest efficacy of 31.2% [11]. The conformational differences between the purified monomeric gp120 protein and its native form could explain these failures. There are evidences to support the fact that a trimeric gp120 is far more superior than monomers in eliciting neutralizing antibodies even though monomeric gp120 capable of inducing neutralizing antibodies have been reported [12–14]. However, a major drawback in evaluating the best immunogen is the time and complexity involved in identifying those candidates that resemble the native form of the gp120 protein. Some of the recently identified bnAbs, which bind exclusively to gp120 trimer, hold the key for rapid screening of potent vaccine candidates [13, 15]. Among these, PG9 and PG16, glycan dependent immunoglobulins isolated from an African donor, recognize an epitope on the quaternary structure of the gp120 protein [15, 16].

Although neutralizing antibodies against gp120 are crucial, an equally important aspect is the generation of HIV-1-specific T-cell immune responses. There is substantial evidence pointing out that HIV-1-specific CD4^+^ and CD8^+^ T-cells mediate protection *in vivo* [17, 18]. An understanding of the crucial role played by T-cells in HIV-1 suppression comes from studying the immune system in “Elite controllers”, a group of people who are able to control HIV-1 replication without any treatment [19, 20]. Of the numerous clinical trials carried out so far with different HIV/AIDS vaccine candidates, only the RV144 phase III clinical trial based on priming with a recombinant canarypoxvirus ALVAC expressing the Env protein and boosting with an adjuvanted monomeric HIV-1 gp120 protein showed a modest protection of 31.2% [11]. The induction of high affinity IgG antibodies against V1/V2 and V3 regions of gp120 was determined to be an important correlate of reduced risk in this study [21–25]. Although the percentage of protection was low, this trial opened up new avenues for developing an effective vaccine against HIV/AIDS. Therefore, improving the immunogenicity of gp120 protein to provide a balanced humoral and T-cell immune response could be of help in the development of a successful gp120-based HIV/AIDS vaccine.

We have previously described a procedure to generate oligomeric forms of the Plasmodium circumsporozoite (CS) protein after its fusion to the VACV 14K (derived from A27L gene) protein and its adjuvant-like effect in prime-boost immunization protocols conferring protection after challenge with the malaria parasite [26]. VACV 14K protein is composed of 110 amino acid (aa) residues containing a heparin binding domain (HBD), a coiled-coil domain (CCD) and a leucine zipper domain (LZD). The HBD (aa 21–34), includes the core sequence KKPE (aa 26–29), which is structurally flexible and essential for binding to cell surface heparan sulfate (HS) [27–29]. The CCD (aa 43–84) is required for self-oligomerization *in vitro* and contains cysteines 71 and 72 to form disulfide bonds during A27 (14K) self-assembly. The LZD (aa 85–110) is the A17 binding region and was predicted to be a leucine zipper. The trimer structure of a truncated form (aa 21–84) of the VACV 14K protein has been defined consisting of two parallel α-helices and one antiparallel α-helix [30]. In the present study, we demonstrate that fusing VACV 14K protein to the C-terminus of HIV-1 gp120 enhanced the immunogenic characteristics of gp120. This HIV-1 fusion protein (termed gp120-14K) was easily purified from mammalian cell cultures and was recognized by a panel of well-known HIV-1 neutralizing antibodies. Furthermore, the HIV-1 gp120-14K immunogen upregulated in human moDCs, proinflammatory cytokines and chemokines associated with a Th1 innate immune response and triggered in immunized mice HIV-1-specific humoral and cellular immune responses. Thus, the gp120-14K protein can be used to enhance the HIV-1-specific T-cell and B-cell immune responses, and might be considered as an HIV-1 immunogen for improved vaccines against HIV/AIDS.

## Materials and Methods

### Ethics Statement

All animal procedures were approved by the Ethical Committee of Animal Experimentation of Centro Nacional de Biotecnologia (CEEA-CNB), in accordance with national and international guidelines and with the Royal Decree (RD 1201/2005). Permit number: 11044.

Studies with peripheral blood mononuclear cells (PBMCs) from healthy blood donors recruited by the “Centro de Transfusión de la Comunidad de Madrid” (Madrid, Spain) were approved by the Ethical Committee of Centro de Transfusión de la Comunidad de Madrid (Madrid, Spain). Written informed consent was obtained from each donor before blood collection, for the purpose of this investigation according to a collaborative agreement between the “Centro de Transfusión de la Comunidad de Madrid” and the CNB-CSIC. All information was kept confidential.

### Cells and viruses

Established chick DF-1 cells (a spontaneously immortalized chicken embryo fibroblast cell line. ATCC, Manassas, VA) and primary chicken embryo fibroblast (CEF) cells [31] were grown in Dulbecco´s modified Eagle´s medium (DMEM) supplemented with 10% heat-inactivated fetal calf serum (FCS), as previously described [31]. MoDCs were obtained as previously described [32–34]. Briefly, peripheral blood mononuclear cells (PBMCs) from buffy coats of healthy donors (recruited by the Centro de Transfusión de la Comunidad de Madrid, Madrid, Spain) were obtained by Ficoll gradient separation on Ficoll-Paque (GE Healthcare). Then, CD14^+^ monocytes were purified by negative selection using Dynabeads Untouched human monocytes kit (Invitrogen Dynal AS, Oslo, Norway), following manufacturers protocol. Next, to obtain moDCs, purified monocytes were cultured for 7 days in 6-well plates (3 × 10^6^ cells/well at 1×10^6^ cells/ml) in complete RPMI 1640 medium containing 10% heat-inactivated FCS and supplemented with 50 ng/ml granulocyte-macrophage colony-stimulating factor (GM-CSF) and 20 ng/ml IL-4 (both from Gibco-Life Technologies). 293T cells were maintained at appropriate conditions as described by ATCC. Stable transfected mammalian Chinese Hamster Ovary (CHO) cells (CHO-K1 and CHO-Lec_3.2.8.1_ cells) [35] expressing HIV-1 gp120-14K were grown in roller bottles with MEM medium lacking glutamine in the presence of 25 μM of the negative selective agent L-methionine sulfoximine (Sigma-Aldrich) and supplemented with 3% FCS. Cell cultures were maintained at 37°C (CEF, moDCs, 293T and CHO) or 39°C (DF-1) in a humidified incubator containing 5% CO_2_.

The poxvirus strains used in this work included the attenuated MVA-WT and the recombinant MVA-B expressing the HIV-1 gp120 protein, as a cell-released product, and HIV-1 Gag-Pol-Nef as an intracellular polyprotein from HIV-1 clade B isolates [31–34, 36–38]. All viruses were grown in primary CEF cells, purified by centrifugation through two 36% (w/v) sucrose cushions in 10 mM Tris-HCl pH 9, and titrated in DF-1 cells by plaque immunostaining assay, as previously described [31]. The titer determinations of the different viruses were performed at least two times. All viruses were free of contamination with mycoplasma, fungi or bacteria.

### Construction and purification of plasmid DNA vectors

Different plasmid DNA vectors were used in this study, such as pCMV-gp120_BX08_, pcDNA-gp120-14K and pBJ5-GS-gp120-14K. A) pCMV-gp120_BX08_: Plasmid vector expressing the mammalian codon optimized gp120 of HIV-1_BX08_ isolate from clade B, containing an artificial signal peptide for enhanced secretion of the protein; the gp120 encoding BX08 gene was kindly provided by Sanofi-Pasteur. B) pcDNA-gp120-14K: Plasmid expressing the mammalian codon optimized gp120 of HIV-1_BX08_ isolate from clade B, fused to the VACV 14K protein (derived from A27L gene), was generated following a similar strategy to the one previously described [26]. C) pBJ5-GS-gp120-14K: The chimeric HIV-1 gp120-14K gene was cloned into pBJ5-GS plasmid, a kind gift from José Casasnovas (CNB-CSIC), which contains a glutamine synthetase minigene. This plasmid was used to generate the stable CHO cell lines (CHO-K1 and CHO-Lec) expressing HIV-1 gp120-14K.

All plasmids were purified using EndoFree Plasmid Mega Kit according to the manufacturer’s instructions (Qiagen) and their correct generation and expression was confirmed by DNA sequence analysis (Secugen, Spain) and western blot using specific antibodies.

### Protein purification

HIV-1 gp120-14K proteins were purified from the CHO-K1 and CHO-Lec cell supernatants by affinity chromatography with *Galanthus nivalis* lectin columns (Vector Labs), to obtain HIV-1 gp120-14K_CHO-K1_ and gp120-14K_CHO-Lec_, respectively. In the case of HIV-1 gp120_BX08_ protein, 293T cells grown in 150 mm plates were transfected using standard calcium phosphate method [39] with 50 μg of the plasmid vector pCMV-gp120_BX08_ per plate and after 72 h, gp120 was purified from clarified cell supernatants through the lectin column. A liter of clarified cell supernatants were pumped through the phosphate-buffered saline (PBS)-prewashed column at a rate of 0.2 ml/min using a peristaltic pump. The column was then washed with 15 ml of cold PBS, and the proteins were eluted with 25 ml of 0.5 M methyl-α-D-manno-pyrannoside (Sigma) at a rate of 0.2 ml/min. Positive fractions were pooled and then passed through a Superdex-200 size exclusion chromatogram (SEC) according to manufacturer’s instructions (GE Healthcare). The fractions containing the oligomers were pooled and then concentrated using centrifugal concentrators with a cutoff of 100 kDa (Millipore). Furthermore, gp120-14K protein was also purified using a monoclonal antibody generated in our laboratory, which has high affinity for the gp120-14K oligomer but not the monomer (data not shown). Moreover, gp120-14K protein purified from CHO-Lec cells was further deglycosylated by treatment with Endo-H, according to manufacturer’s instructions (Sigma Aldrich). The proteins were tested for LPS contamination using chromogenic Limulus Amebocyte Lysate (LAL) kit (QCL-1000, Lonza). Proteins remained stable at room temperature (RT) for more than 2 weeks and had less than 0.2 endotoxin units (EU) of LPS per mg of protein based on LAL assay (Lonza). The purified clade B HIV-1 gp120 protein from isolate IIIB (EVA607) was obtained from the Centre for AIDS Reagents, NIBSC and was donated by ImmunoDiagnostics Inc, and the purified gp140 clade B consensus (B.con_env03 gp140 CF) was a kind gift of Barton Haynes (Duke University). The purified clade C HIV-1 gp140 (from isolate CN54) was a kind gift from Greg Spies (Fred Hutchinson Cancer Research Center).

### SDS-PAGE and blue native (BN) PAGE

The recombinant HIV-1 gp120-14K proteins were analyzed by SDS-PAGE and BN-PAGE and stained with coomassie blue. Briefly, the proteins were mixed with 1X Laemmli buffer and run on a 10% SDS-PAGE with or without β-mercaptoethanol (reducing and non-reducing conditions, respectively). For BN-PAGE the NativePAGE Novex Bis-Tris gel system (Invitrogen) was used according to manufacturer’s instructions.

### Electron microscopy and image processing

Samples of HIV-1 gp120-14K were applied onto carbon-coated copper grids and stained with 2% uranyl acetate. Micrographs were taken under minimal dose conditions in a JEOL JEM1200EXII microscope operated at 100 kV and digitized in a *Nikon* Super *CoolScan 9000* scanner with a pixel size of 2.12 Å/pixel. Individual particles were manually selected using XMIPP3.1 [40]. Image classification was performed using multi-reference free pattern based on correlation and standard maximum correlation criterion refinement (CL2D), as implemented in XMIPP3.1 [41]. Homogeneous populations were obtained and averaged for a final two-dimensional characterization.

### RNA extraction and quantitative RT-PCR

Total RNA was isolated from moDCs (2×10^5^ cells) mock-treated or treated with 5 μg of HIV-1 gp120 and gp120-14K_CHO-K1_ proteins in a 96-well plate, using RNeasy Kit (Qiagen) according to manufacturer’s instruction. cDNA was obtained from 1 μg of RNA using QuantiTect Reverse Transcription kit (Qiagen). Quantitative real-time PCR was carried out with a 7500 Real-Time PCR system (Applied Biosystems) using the Power SYBR Green PCR Master Mix (Applied Biosystems), as previously described [33, 42]. Expression levels of different genes involved in innate immune responses (IL-1β, IFN-γ, IL-8, IL-12, IL-6 and RANTES) were analyzed at 3 and 6 h post-treatment. The expression levels were represented as arbitrary units (AU) with reference to the house keeping gene hypoxanthine guanine phosphoribosyltransferase (HPRT). All samples were tested in duplicate, and two independent experiments were performed.

### Chemokine measurements by Luminex

IL-6 and RANTES concentrations in cell-culture supernatants from moDCs (2×10^5^ cells) mock-treated or treated with 5 μg of HIV-1 gp120 and gp120-14K_CHO-K1_ proteins were measured at 24 h post-treatment using Luminex technology according to manufacturer’s instructions (Millipore).

### Animals and immunizations

Female BALB/c mice (H-2^d^), 6–8 weeks old, were obtained from Harlan Laboratories. Different homologous or heterologous prime/boost immunization protocols were performed to assay the immunogenicity of the HIV-1 gp120-14K fusion protein. In summary, animals (n = 8 per group) were immunized with 100 μg of DNA (DNA-gp120, DNA-gp120-14K or DNA-ϕ), or 20 μg of protein (gp120-14K_CHO-K1_ or gp120-14K_CHO-Lec_) via intradermal (i.d.) route or with 2 x 10^7^ PFU of MVA-B virus via intraperitoneal (i.p.) route. Two weeks later, animals were boosted with 2 x 10^7^ PFU of MVA-WT or MVA-B through i.p. injection or with 20 μg of gp120-14K_CHO-K1_ protein via i.d. route. All the preparations were made in endotoxin free PBS. Following immunization, animals were sacrificed on day 10 and 68 using carbon dioxide (CO_2_), and their spleens were processed to measure the adaptive and memory immune responses, respectively. Two independent experiments were performed.

### Multiparameter flow cytometry

The magnitude, polyfunctionality and phenotypes of the HIV-1-specific T-cell adaptive and memory responses were analyzed by flow cytometry and intracellular cytokine staining (ICS) as previously described [33, 43]. Briefly, splenocytes were rested overnight and the following day 4×10^6^ splenocytes were stimulated with 5 μg/ml of HIV-1 Env peptide pool spanning the full length gp120 from BX08 isolate in addition to 1 μl/ml GolgiPlug (BD Biosciences), anti-CD107a-Alexa 488 (BD Biosciences), and monensin (1X; eBioscience) in RPMI 1640 media supplemented with 10% FCS for 6 h in a 96 well plate. Peptides were provided by the Eurovacc Foundation and were previously described [31]. Also, splenocytes were stimulated with A20 cells transfected with DNA-gp120 (4 x 10^5^ gp120 transfected A20 cells in 4 x 10^6^ splenocytes; the ratio of gp120-transfected A20 cells to splenocytes was equal to 1:10). Following stimulation, cells were washed, Fc receptors were blocked using anti CD16/CD32 (BD Biosciences), stained for the surface markers, fixed, permeabilized (Cytofix/Cytoperm kit; BD Biosciences), and stained intracellularly for cytokines with the appropriate fluorochromes. Dead cells were excluded using the violet LIVE/DEAD stain kit (Invitrogen). Cells were stained with different mouse antibodies, such as CD3-PE-CF594, CD4-APC-Cy7, CD8-V500, IFN-γ-PE-Cy7, IL-2-APC and TNF-α-PE (all from BD Biosciences). In addition, for differentiating memory T-cells the following antibodies were used: CD62L-Alexa 700 (BD Biosciences) and CD127-PerCP-Cy5.5 (eBioscience). A million cells were then passed through GALLIOS flow cytometer (Beckman Coulter) and the data was analyzed with FlowJo (Tree Star. Inc) and Spice (version 5.0). Appropriate controls were used and the values from unstimulated samples were subtracted.

### ELISA analysis

Antibodies present in the serum of immunized animals were determined using ELISA as previously described [26, 33, 37]. Human broadly neutralizing antibodies (bnAbs) PG9 (ARP3294), PG16 (ARP3293), b12 (EVA3065), VRC01 (ARP3291) and 2G12 (EVA3064) were obtained from the Centre for AIDS Reagents, NIBSC. Human antibodies PGT-121, 257-D IV were obtained from the NIH AIDS Reagent Program, Division of AIDS, NIAID, NIH (USA). Human antibody 10–1074 was a kind gift of Michel Nussenzweig (Rockefeller University). Proteins tested for binding to human bnAbs were: gp120-14K_Lectin/SEC_ protein, derived from CHO-K1 cells purified by lectin columns followed by SEC; gp120-14K_Ab purified_ protein, purified using a monoclonal antibody generated in our laboratory which has high affinity for the gp120-14K oligomer but not the monomer; gp120-14K_Deglycosylated_ protein, purified from CHO-Lec cells and treated with Endo-H, and recombinant clade B gp120 protein (IIIB). Other proteins from clades B and C used for binding to human bnAbs were: gp120 (BX08); gp140 (B consensus) and gp140_CN54_ (clade C). Purified proteins were coated on 96 well Nunc Maxisorp plates at a concentration of 2 μg/ml in PBS and incubated at 4°C overnight. Serial dilutions of Abs were made and the bound antibodies to Env were detected using 1:2000 dilution of HRP conjugated goat anti-human antibody (Sigma). Furthermore, antibody levels in vaccinated animals against proteins gp120-IIIB, gp120-14K_CHO-K1_ and PYCS-14K (an oligomeric fusion protein between circumsporozoite protein of malaria parasite *Plasmodium yoelii* and 14K) were detected using 1:2000 dilution of HRP conjugated goat anti-mouse antibody total IgG, IgG1, IgG2a or IgG3 (Southern Biotechnology Associated, Birmingham). Plates were developed by adding 3,3’,5,5’ Tetramethylbenzidine (TMB) substrate (Sigma) and stopping the reaction with 1 M H_2_SO_4_. Absorbance was read at 450 nm. Endpoint titer values were determined as the last positive dilution of serum giving an absorbance value three times higher than naïve serum.

### Data analysis

Statistical analysis was performed using Minitab for Windows. Unpaired Student´s T test was performed to compare responses between groups. ELISA endpoint titers were logarithmically transformed before comparison. For ICS, statistical analysis was done based on previously described method [37, 44].

## Results

### Production and purification of HIV-1 gp120-14K protein

**Fig 1:**
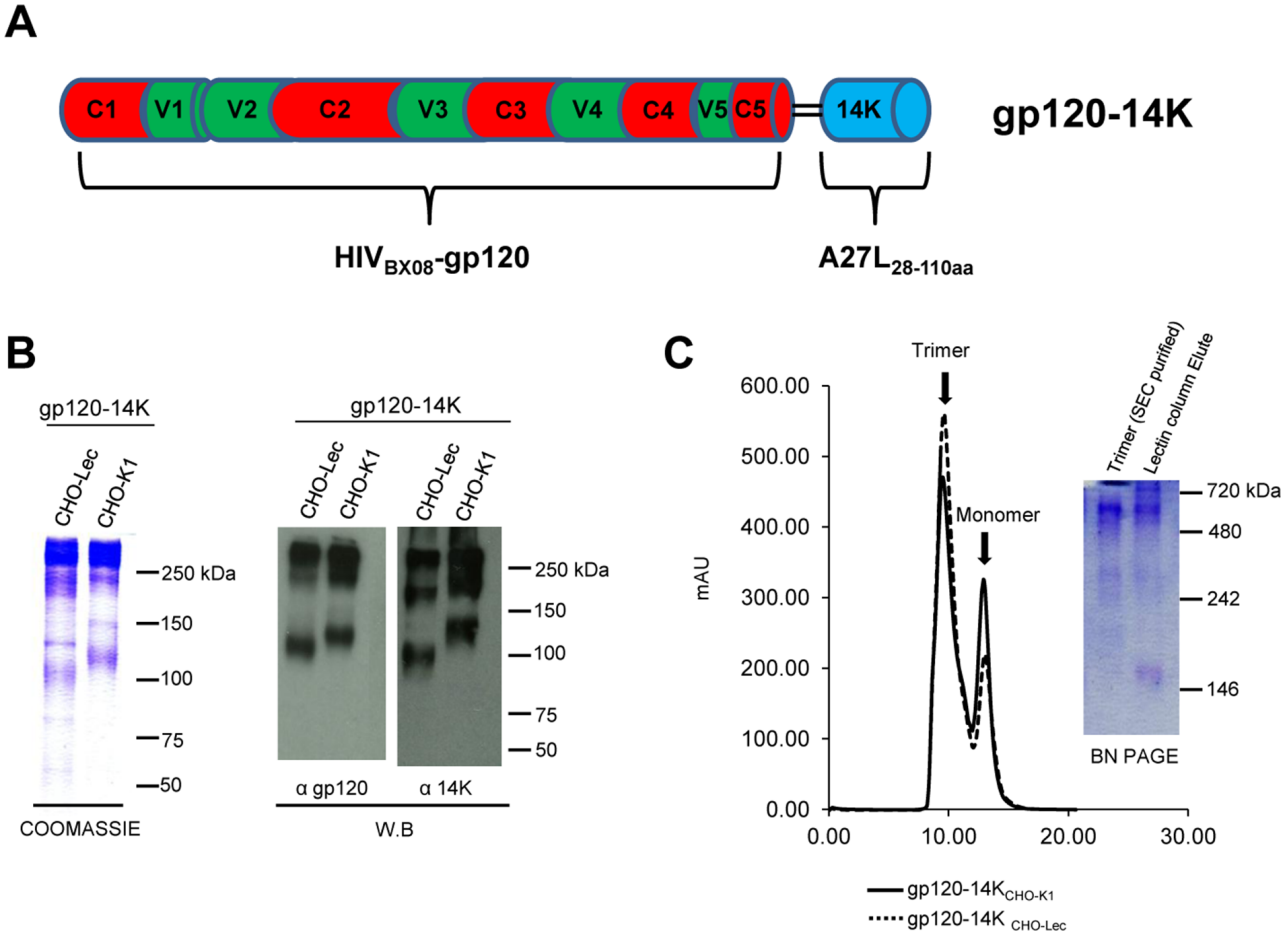
Characterization of recombinant HIV-1 gp120-14K fusion proteins. **(A)** Schematic representation of a codon optimized HIV-1 gp120_BX08_ fused with VACV A27L gene lacking the first 28 aa. **(B)** SDS-PAGE of gp120-14K purified proteins under non-reduced condition detected by coomassie staining or western blotting (W.B.) using a rabbit derived polyclonal antibody against gp120 (Centro Nacional de Biotecnologia, 1:3000) and mouse monoclonal antibody against VACV 14K protein (Centro Nacional de Biotecnologia, 1:1000). **(C)** SEC profile of lectin column purified gp120-14K protein from CHO-K1 or CHO-Lec cells using Superdex 200 column. BN-PAGE comparative analysis of SEC purified gp120-14K_CHO-K1_ and lectin column eluted protein.

**Fig 2:**
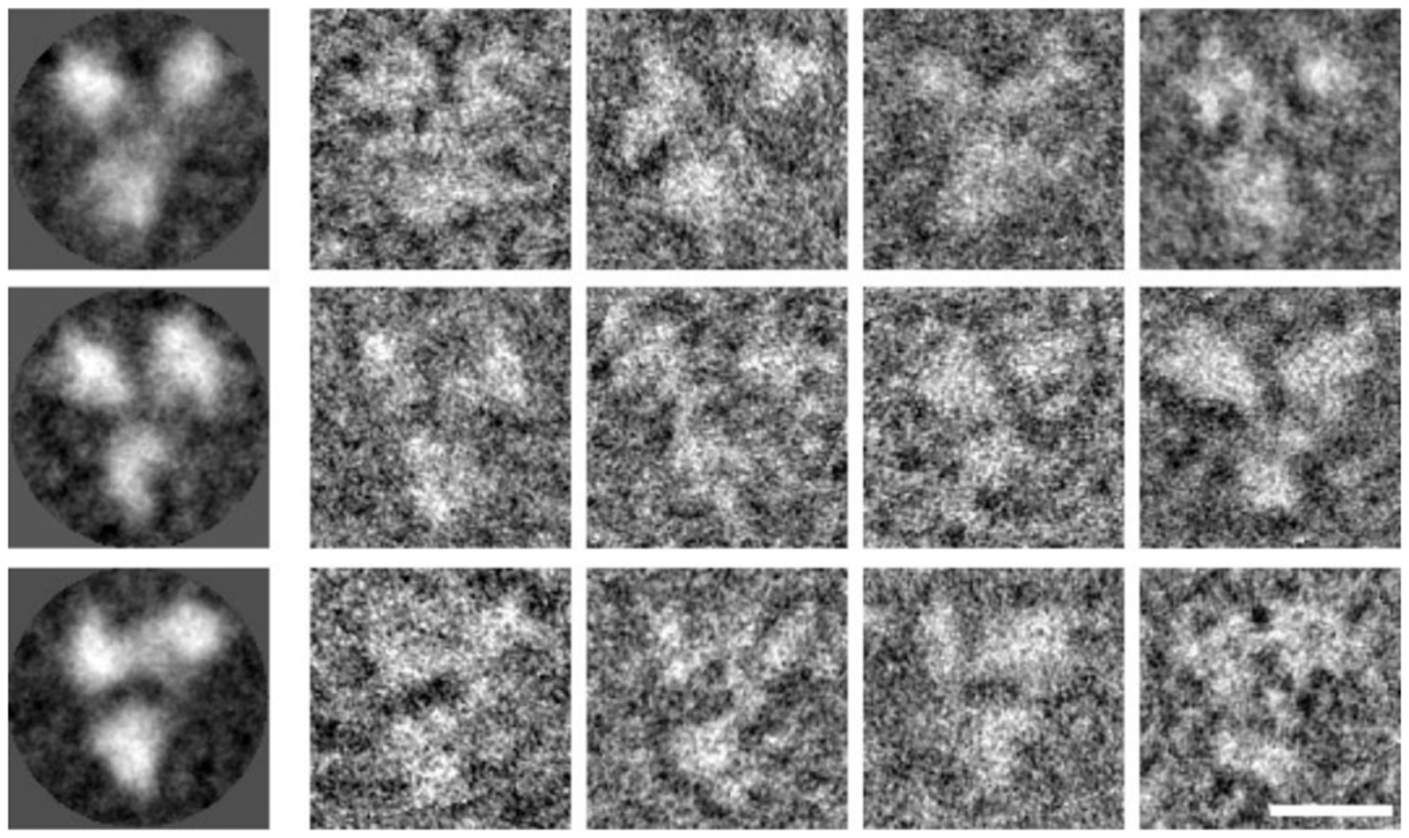
Transmission electron microscopy of negatively stained HIV-1 gp120-14K particles. Panel on the left shows averaged images corresponding to different top views of SEC purified gp120-14K particles. Panels on the right show several individual views of gp120-14K particles belonging to the averaged classes in the left column. Scale bar corresponds to 10 nm.

To produce an oligomeric HIV-1 gp120, we took advantage of the 110 aa of the VACV 14K protein (derived from A27L gene) that forms trimeric coiled coils [30], and forms oligomers when fused to the Plasmodium circumsporozoite CS antigen [26]. Thus, a N-terminal truncated VACV 14K protein (1–28 aa, to remove the GAG binding domain) was fused to the C-terminus of a clade B HIV-1 gp120 protein (from isolate BX08), generating the HIV-1 gp120-14K protein (Fig 1A), in a similar fashion to what we reported previously for a malaria antigen [26]. The clade B HIV-1 gp120 protein used in this study is a codon optimized synthetic construct derived from isolate BX08 (AAG49242.1), which has 92% and 88% similarity with optimized HIV-1 subtype B consensus gp120 (ABG67916.1) and with clade B HIV-1 gp120 from IIIB isolate (AIJ50275.1), respectively. For stable and large scale production, we generated CHO cell lines expressing HIV-1 gp120-14K protein, as described under Materials and Methods. Proteins were purified using *Galanthus nivalis* lectin from the supernatants of CHO-K1 and CHO-Lec_3.2.8.1_ (a glycosylation defective mutant cell line) cell lines with a yield of approximately 7 mg/liter. In addition, we purified the protein using a monoclonal antibody which has high affinity for the gp120-14K oligomer but not for the monomer. The purified HIV-1 gp120-14K proteins were run on SDS-PAGE under non-reducing conditions and the corresponding coomassie stained gels and western blots using antibodies against HIV-1 gp120 or VACV 14K proteins showed that most gp120-14K proteins appear as high molecular weight (Fig 1B), with subtle differences observed in the size of monomers derived from CHO-K1 (gp120-14K_CHO-K1_) versus CHO-Lec (gp120-14K_CHO-Lec_) cells, probably due to different glycosylation or mannose content. Moreover, the SEC profile of HIV-1 purified proteins from CHO-K1 and CHO-Lec cells reveals the presence of HIV-1 gp120-14K, mainly as oligomers over monomers (Fig 1C, left panel). The oligomeric fraction of SEC purified gp120-14K_CHO-K1_ was further analyzed by BN-PAGE (Fig 1C). The main band of about 520 kDa in the pooled oligomeric fraction was compatible with a trimer (Fig 1C, lane 1). The BN-PAGE also reveals a minor presence of monomers over oligomers in the purified gp120-14K_CHO-K1_ proteins before SEC (Fig 1C, lane 2). Furthermore, the state of individual particles of HIV-1 gp120-14K was evaluated by electron microscopy, which clearly highlights the three lobes of HIV-1 gp120 indicating a trimeric assembly (Fig 2).

Overall, we successfully purified an oligomeric HIV-1 gp120-14K protein and established a viable cell system capable of stable expression of gp120-14K proteins in significant quantities.

### Antigenic characteristics of HIV-1 gp120-14K determined by binding to a panel of broadly neutralizing human monoclonal antibodies

Next, we examined the antibody binding characteristics of the HIV-1 gp120-14K protein, which was purified by lectin and SEC (gp120-14K_Lectin/SEC_) or by immunoaffinity (gp120-14K_Ab purified_), against a panel of well characterized HIV-1 neutralizing antibodies, in comparison with purified HIV-1 gp120, gp140 and deglycosylated gp120-14K.

#### Quaternary conformation dependent binding antibodies

**Fig 3:**
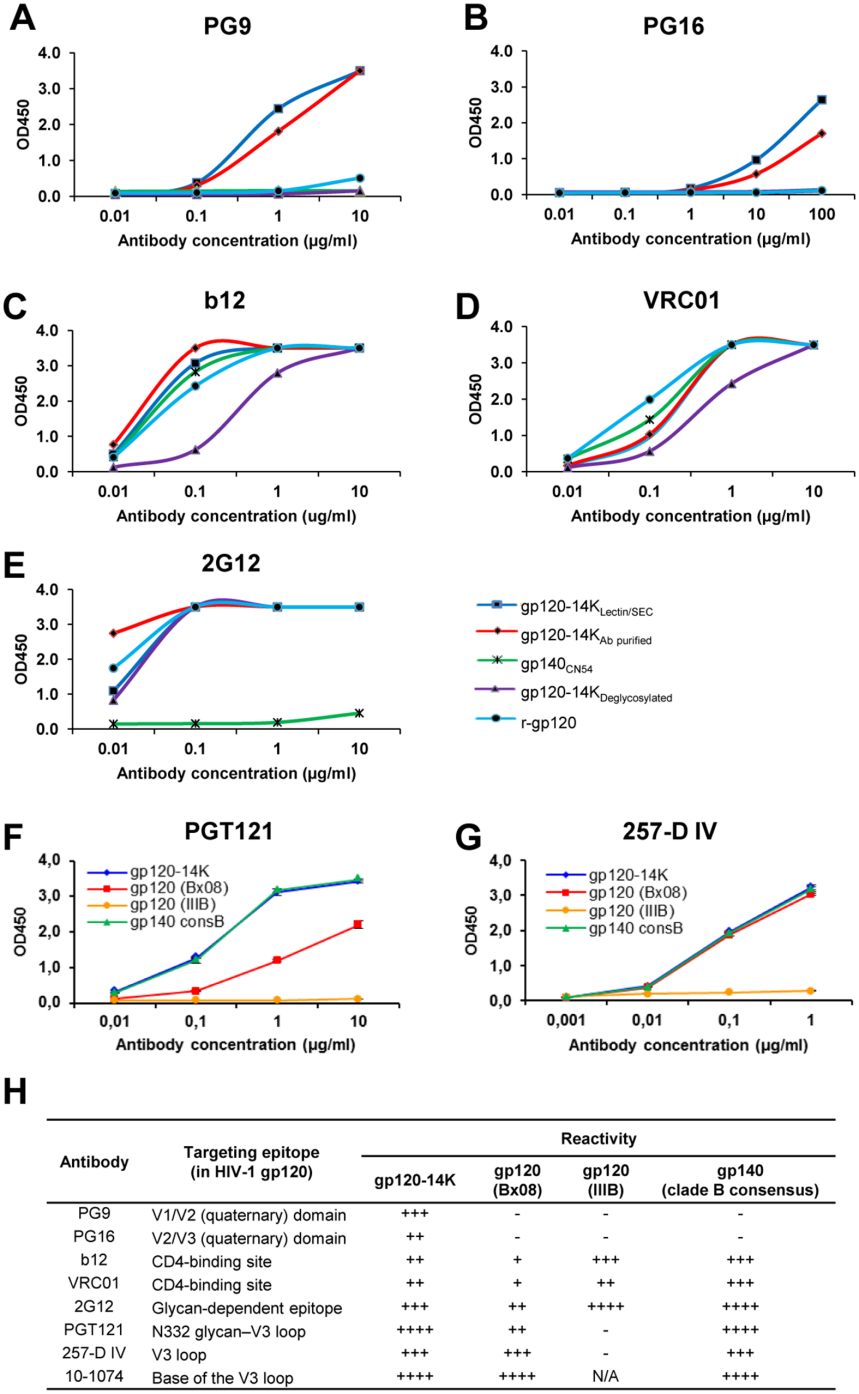
The fused gp120-14K protein is recognized by a broad panel of HIV-1 neutralizing antibodies against gp120. Representative binding curves of different HIV-1 proteins with a panel of well-known neutralizing Abs, such as quaternary conformation dependent antibodies PG9 **(A)** and PG16 **(B)**; bnAbs targeting the CD4 binding site b12 **(C)** and VRC01 **(D)**; the glycan dependent antibody 2G12 **(E)** and antibodies targeting the V3 loop PGT121 **(F)** and 257-D IV **(G)**. **(H)** Table showing the reactivity of HIV-1 gp120-14K, gp120 (BX08), gp120 (IIIB) and gp140 (clade B consensus) proteins against a panel of well-known neutralizing antibodies. The binding level is indicated using plus or minus symbols. N/A: not available. HIV-1 proteins used in panels A to E were: gp120-14K_Lectin/SEC_ protein, derived from CHO-K1 cells purified by lectin columns followed by SEC; gp120-14K_Ab purified_ protein, purified using a monoclonal antibody generated in our laboratory; gp140_CN54_ protein; gp120-14K_Deglycosylated_ protein, purified from CHO-Lec cells and treated with Endo-H; r-gp120 monomeric gp120 protein (IIIB). HIV-1 proteins used in panels F and H were: gp120-14K_Lectin/SEC_, gp120 (BX08), gp120 (IIIB) and gp140 (clade B consensus).

An important class of HIV-1 gp120 neutralizing antibodies recognizes the quaternary structure of gp120, targeting the V2 and V3 loops [45], such as PG9 and PG16, which binds strongly to trimeric gp120 in a glycan dependent fashion [15, 46]. Here, we used PG9 and PG16 antibodies to evaluate the structural conformation of the HIV-1 gp120-14K protein. The results showed that both antibodies bind, although weakly, to gp120-14K protein (Fig 3A and 3B), with PG9 having higher affinity than PG16. These antibodies did not bind with monomeric gp120 or with gp140, as well as with the Endo-H treated gp120-14K_CHO-Lec_ (gp120-14K_Deglycosylated_).

#### CD4 binding antibodies

Antibodies targeting the CD4 binding site (CD4bs) are considered to be of prime importance in HIV-1 neutralization, considering the fact that binding of gp120 to CD4 initiates the mechanism behind viral entry [47, 48]. Among this class of widely studied antibodies, b12 and VRC01 are of paramount significance [49, 50]. Primary analysis based on a capture ELISA with soluble human CD4 showed that HIV-1 gp120 and gp120-14K proteins, irrespective of their conformation or glycosylation, have similar affinities for CD4 (data not shown). The ELISA binding of b12 and VRC01 showed that these antibodies had similar binding capacity to the different gp120 proteins, irrespective of their conformation (Fig 3C and 3D). However, gp120-14K_Deglycosylated_ had lesser affinity to these antibodies. Thus, the conformation of the HIV-1 gp120-14K protein neither occludes the binding of CD4 to the protein nor the antibodies targeting CD4 binding sites.

#### Glycan dependent antibodies

An important class of neutralizing antibodies targets complex sugar molecules on the surface of gp120 [51]. Even though glycans are partly responsible for shielding protective epitopes and are known to neutralize the effect of potent antibodies by glycan repositioning, many potent neutralizing and non-neutralizing antibodies target these sugars [51]. 2G12 is a well-known antibody belonging to this class which binds to the terminal α1→2-linked mannose residues [52]. Thus, we evaluated the binding of varying concentrations of 2G12 antibody to the HIV-1 gp120-14K protein by ELISA, and the results showed that 2G12 binds to both oligomers and monomers of the glycosylated gp120-14K protein and the monomeric gp120 (Fig 3E).

#### V3-loop binding antibodies

Another important class of neutralizing antibodies are those directed against the V3 loop of HIV-1 gp120 [53]. Thus, to carry out direct comparison of the antigenic nature of gp120-14K over gp120 from clade BX08, we produced and purified these two proteins from mammalian cell lines. ELISA was performed with PGT121 [54–57], 257-D-IV [58, 59] and 10–1074 [60] antibodies targeting the V3 loop. PGT121 has higher binding to gp120-14K and gp140 consB than monomeric gp120 (BX08), lacking reactivity with gp120 (IIIB) (Fig 3F). In the case of the V3 loop antibody 257-D IV, there was similar reactivity for gp120-14K as for gp120 (BX08) (Fig 3G). Similar findings were observed with the antibody 10–1074 reacting at the base of the V3 loop (Fig 3H).

Overall, the binding of a panel of HIV-1 neutralizing antibodies (summarized in Fig 3H) reveal that gp120-14K has broad antigenicity as is recognized by a wide spectrum of HIV-1 neutralizing antibodies.

### HIV-1 gp120-14K upregulates Th1 innate immune responses in human moDCs

**Fig 4:**
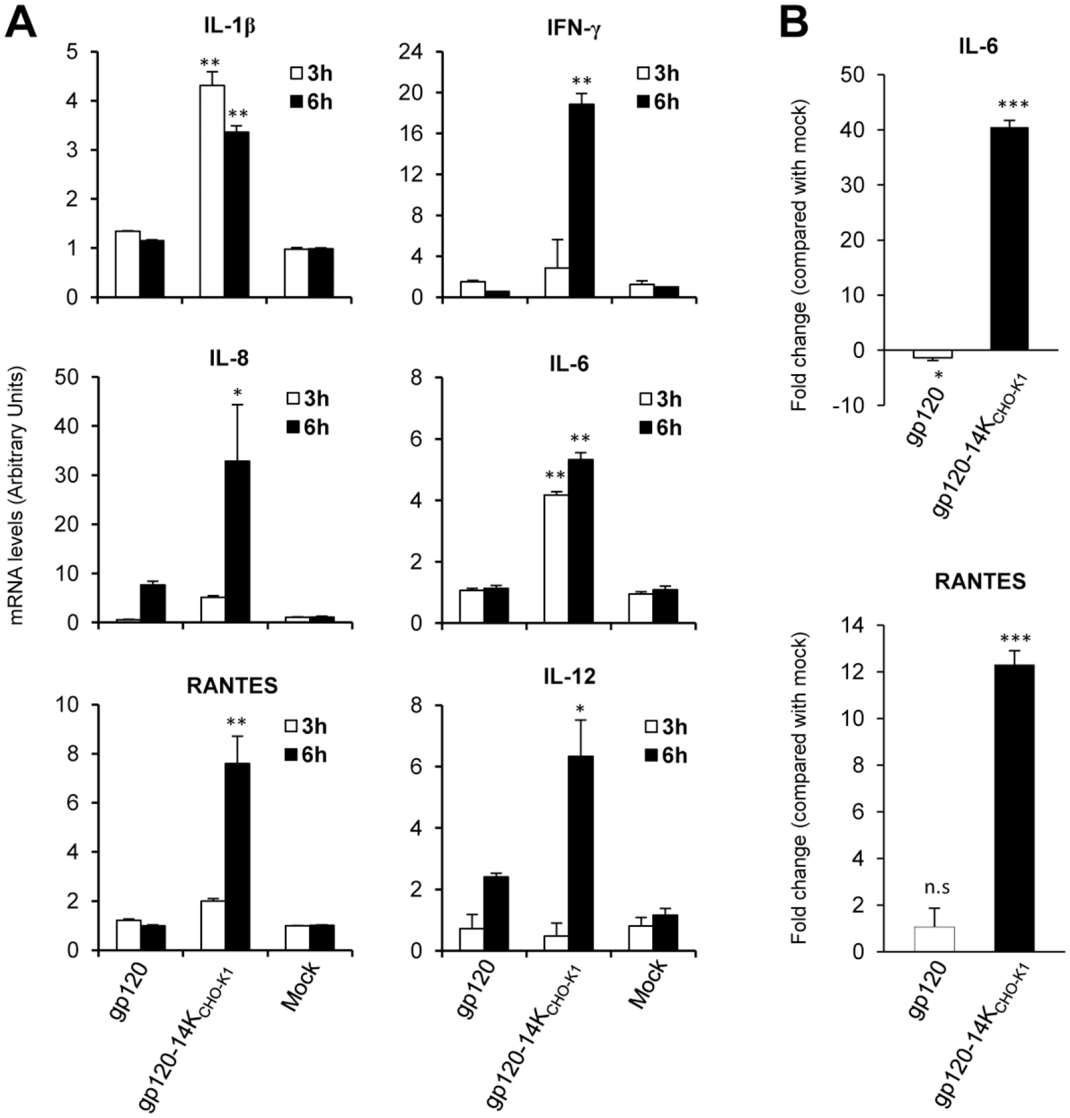
Upregulation of T_H_1 innate immune responses in moDCs by HIV-1 gp120-14K protein. HIV-1 gp120-14K protein (5μg), derived from CHO-K1 cells, was used to analyze the activation of innate immune signaling in human moDCs at 3 and 6 hours. For comparison gp120-IIIB protein (1μg) was used. (A) Quantitative RT-PCR analysis of genes involved in innate immune signaling. 1μg of RNA extracted from protein-treated moDCs, were converted into cDNA and 2μl of cDNA was used as starting material for qRT-PCR according to manufacturer’s instructions. Results were expressed as the ratio of gene to HPRT mRNA levels. AU: arbitrary units. p values indicate significantly higher responses comparing gp120-14K to gp120 at the same hour. * p<0.05, ** p<0.005. Data are means ± SD of duplicate samples and are representative of two independent experiments. (B) Luminex assay. The levels of IL-6 and RANTES in the supernatant from moDCs stimulated with the gp120 and gp120-14K proteins were evaluated after 24 hours. The differences are represented as fold change when compared with mock treated cells. Data are expressed as mean ± SD of triplicates from two different donors. p values indicate significantly higher responses comparing gp120-14K and gp120 to mock-treated cells. * p<0.05, *** p<0.001.

Innate immune sensing of proteins by DCs plays a pivotal role in shaping up long-term immune responses [61]. Therefore, we examined the profiling of innate immune responses induced in human moDCs stimulated with monomeric gp120 and SEC purified oligomeric gp120-14K_CHO-K1_ proteins, by using quantitative RT-PCR and Luminex. The results showed that genes influencing Th1 innate immune responses, such as IL-1β, IL-8, RANTES, IFN-γ, IL-6 and IL-12p40 were significantly upregulated by gp120-14K_CHO-K1_ at 3 and 6 h post-treatment, in comparison to mock cells or to monomeric gp120 protein (Fig 4A). Furthermore, upregulation of pro-inflammatory cytokines IL-6 and RANTES at mRNA level, by treatment with gp120-14K_CHO-K1_, were validated using Luminex, analyzing the supernatants from moDCs at 24 h post-treatment (Fig 4B). Thus, these data demonstrated the ability of HIV-1 gp120-14K to upregulate the innate immune responses, favoring a Th1 response in human moDCs.

### Vaccine based on HIV-1 gp120-14K induces polyfunctional Env-specific CD4^+^ and CD8^+^ T-cell adaptive and memory immune responses

**Fig 5:**
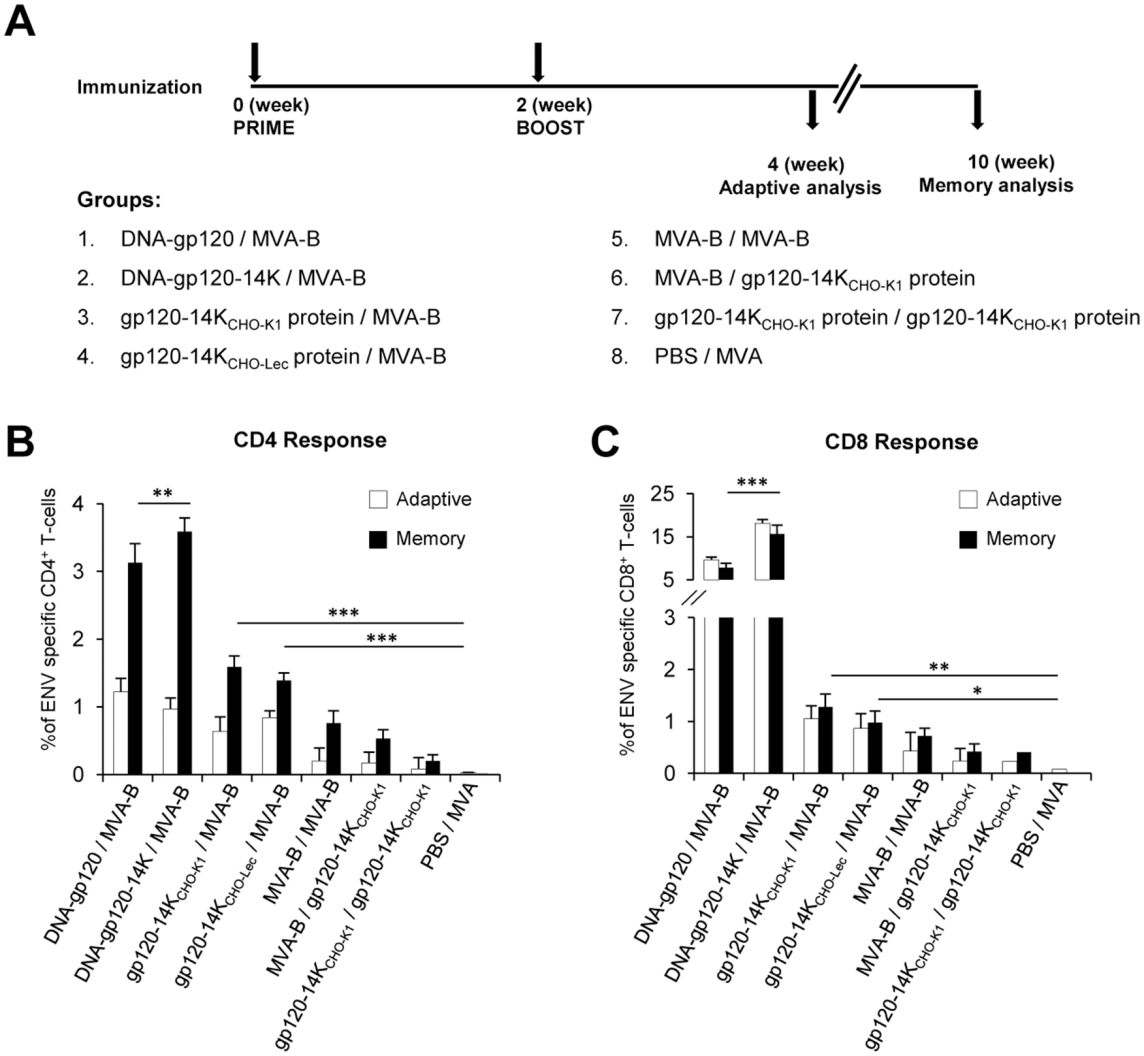
Vaccination regimen based on DNA-gp120-14K priming and MVA-B boosting elicits high frequency of gp120-specific T-cells. **(A)** Immunization schedule of animals. Balb/c mice were inoculated with 100 μg of respective plasmids or 20 μg of proteins intradermally or with 2×10^7^ PFU of recombinant MVA virus expressing gp120 and Gag-Pol-Nef of HIV clade B virus (MVA-B) via intraperitoneal route. The total Env-specific CD4^+^ **(B)** and CD8^+^ (**C)** T-cell responses induced by the vaccination during adaptive and memory phases were measured by stimulating the splenocytes with overlapping peptides for gp120, as described in Materials and Methods. Data are representative of two independent experiments. * p < 0.05; ** p < 0.005; *** p < 0.001.

The role of Env-specific CD4^+^ and CD8^+^ T-cells are well documented in controlling viral growth [17]. This was further substantiated by studies in macaques which ratified the importance of CD8^+^ T-cells in reducing viremia [62]. Thus, in this study we evaluated in mice, the immunogenicity of HIV-1 gp120-14K, using different prime/boost combinations with DNA, protein or a recombinant MVA expressing HIV-1 Env, Gag, Pol and Nef antigens from clade B (termed MVA-B). The different groups and the immunization schedules are represented (Fig 5A) and are described in Materials and Methods. There are two immunization groups that can be compared head-to-head to establish the activity of VACV 14K in vivo: groups 1 and 2. Plasmid DNA vectors expressing gp120-14K (DNA-gp120-14K) or gp120 (DNA-gp120) from BX08 were used for priming in this study. The animals received 100 μg of DNA (DNA-gp120 or DNA-gp120-14K) or 20 μg of adjuvant free protein (gp120-14K_CHO-K1_ or gp120-14K_CHO-Lec_) or 2×10^7^ PFU of MVA-WT or MVA-B, either as priming or as boosting agent. DNA and proteins were administered i.d, while viruses (MVA-WT or MVA-B) were given by i.p route. Env-specific CD4^+^ and CD8^+^ T-cell adaptive and memory immune responses induced in immunized mice were measured by ICS.

The total magnitude of Env-specific CD4^+^ and CD8^+^ T-cells during adaptive and memory phases, determined as the sum of the individual responses producing CD107a, IFN-γ, TNF-α and/or IL-2 obtained for the Env peptide pools, was significantly higher in the DNA-gp120-14K/MVA-B immunized animals than in all the other groups (Fig 5B and 5C, respectively). While the total frequency of Env-specific T-cell responses were higher in DNA primed animals than those receiving the gp120-14K protein (gp120-14K_CHO-K1_ or gp120-14K_CHO-Lec_), animals primed with gp120-14K protein and boosted with MVA-B had higher Env-specific CD4^+^ and CD8^+^ T-cell responses than those immunized with the homologous prime/boost protocol with MVA-B, the homologous prime/boost protocol with gp120-14K protein or the heterologous MVA-B prime/gp120-14K boost immunization group (Fig 5B and 5C). Furthermore, overall Env-specific immune responses were mainly mediated by CD8^+^ T cells in DNA-gp120/MVA-B and DNA-gp120-14K/MVA-B immunization groups (Fig 5B and 5C). Moreover, in all the immunization groups, we observed an increment in the magnitude of Env-specific CD4^+^ T-cells during memory phase compared to the adaptive immune responses (Fig 5B). However, magnitudes of Env-specific CD8^+^ T-cells detected were similar during the adaptive and memory phases (Fig 5C). Similar findings were observed in two independent experiments, and comparable results were obtained when A20 cells transfected with DNA-gp120 were used as a stimulus instead of the Env peptides (S1A and S1B Fig).

Based on previous studies on the importance of long-term polyfunctional memory T-cells in mediating protection against viral infections [63], we next analyzed the differences in the polyfunctionality of Env-specific CD4^+^ and CD8^+^ T-cell adaptive and memory immune responses induced by the different immunization protocols. Polyfunctionality was defined by the ability of Env-specific T-cells to express different combinations of CD107a, IFN-γ, TNF-α or IL-2 cytokines, and measures the quality of the T-cell immune responses.

**Fig 6:**
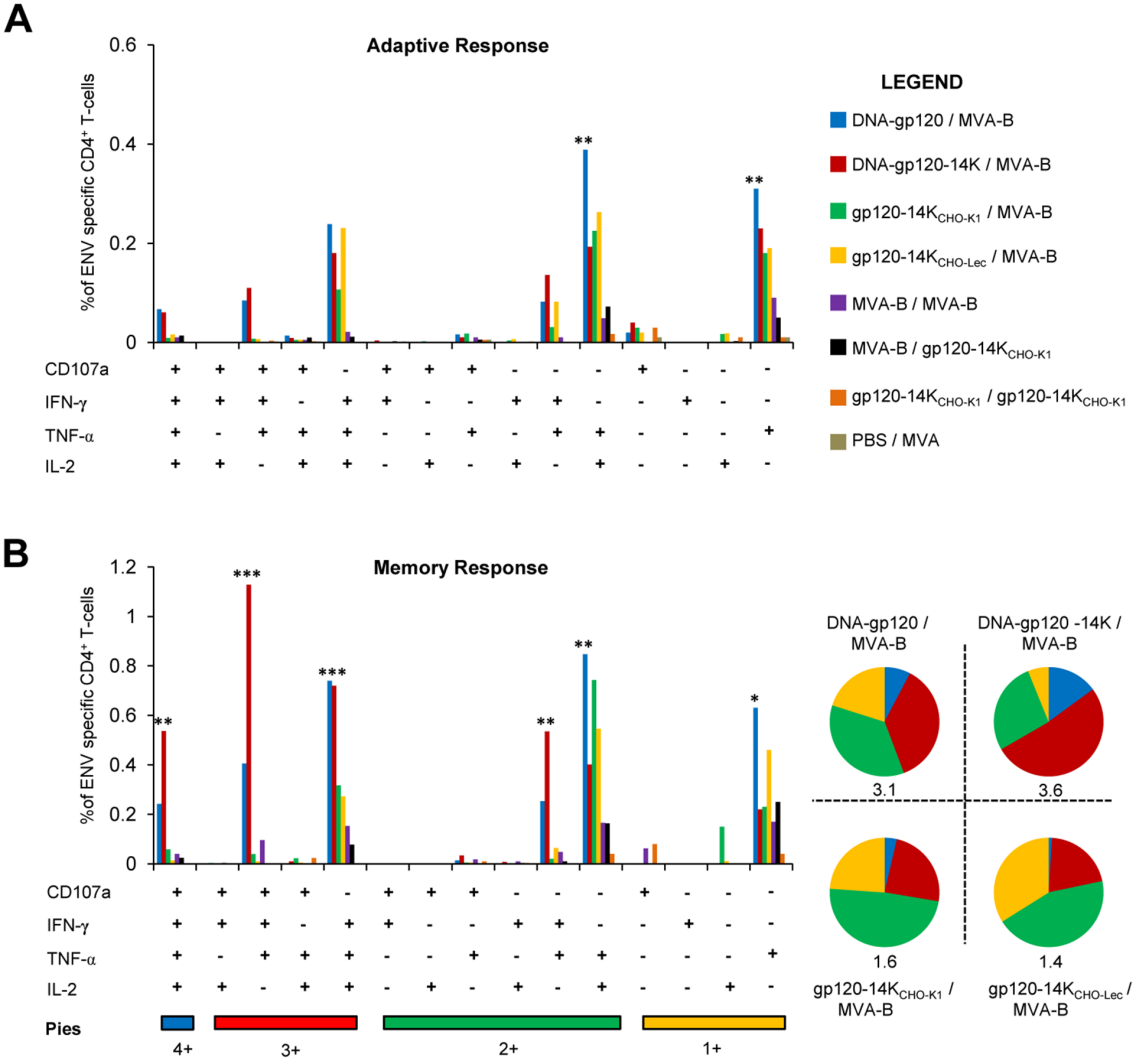
Enhancement of polyfunctional Env-specific CD4^+^ T-cells by HIV-1 gp120-14K. Env-specific CD4^+^ T-cell responses are based on the secretion of CD107a, IFN-γ, TNF-α and IL-2. Polyfunctionality is defined as the ability of the Env-specific CD4^+^ T-cells to secrete a combination of two or more cytokines. **(A)** Polyfunctionality of Env-specific CD4^+^ T-cells in the adaptive phase. **(B)** Polyfunctionality of Env-specific CD4^+^ T-cells in the memory phase. Pie charts represent the distribution of cells secreting different combination of cytokines and numbers below indicate the total magnitude. Data are representative of two independent experiments. Statistical significance between the PBS/MVA control animals and vaccinated animals within the polyfunctional population are represented.* p < 0.05; ** p < 0.005; *** p < 0.001.

The results for the CD4^+^ T cell responses showed that during the adaptive phase, DNA-gp120/MVA-B and DNA-gp120-14K/MVA-B immunization groups induced similar highly polyfunctional profile of Env-specific CD4^+^ T-cells (Fig 6A). However, during the memory phase the polyfunctionality and magnitude of CD4^+^ T-cells was significantly improved by the DNA-gp120-14K/MVA-B immunization group, with nearly a two-fold improvement in the CD107a^+^IFN-γ^+^TNF-α^+^IL-2^+^ population (*p<*0.005) and a 1.4-fold increase in T-cells producing 3 cytokines, with the dominant populations being CD107a^+^IFN-γ^+^TNF-α^+^ and IFN-γ^+^TNF-α^+^IL-2^+^ (*p<*0.001) (Fig 6B). Additionally, while the gp120-14K protein-primed animals produced polyfunctional adaptive and memory CD4^+^ T-cells, the magnitude was significantly lower than those induced by the DNA-primed groups, but significantly higher than those produced by the homologous MVA-B/MVA-B immunization group (*p<*0.005).

**Fig 7:**
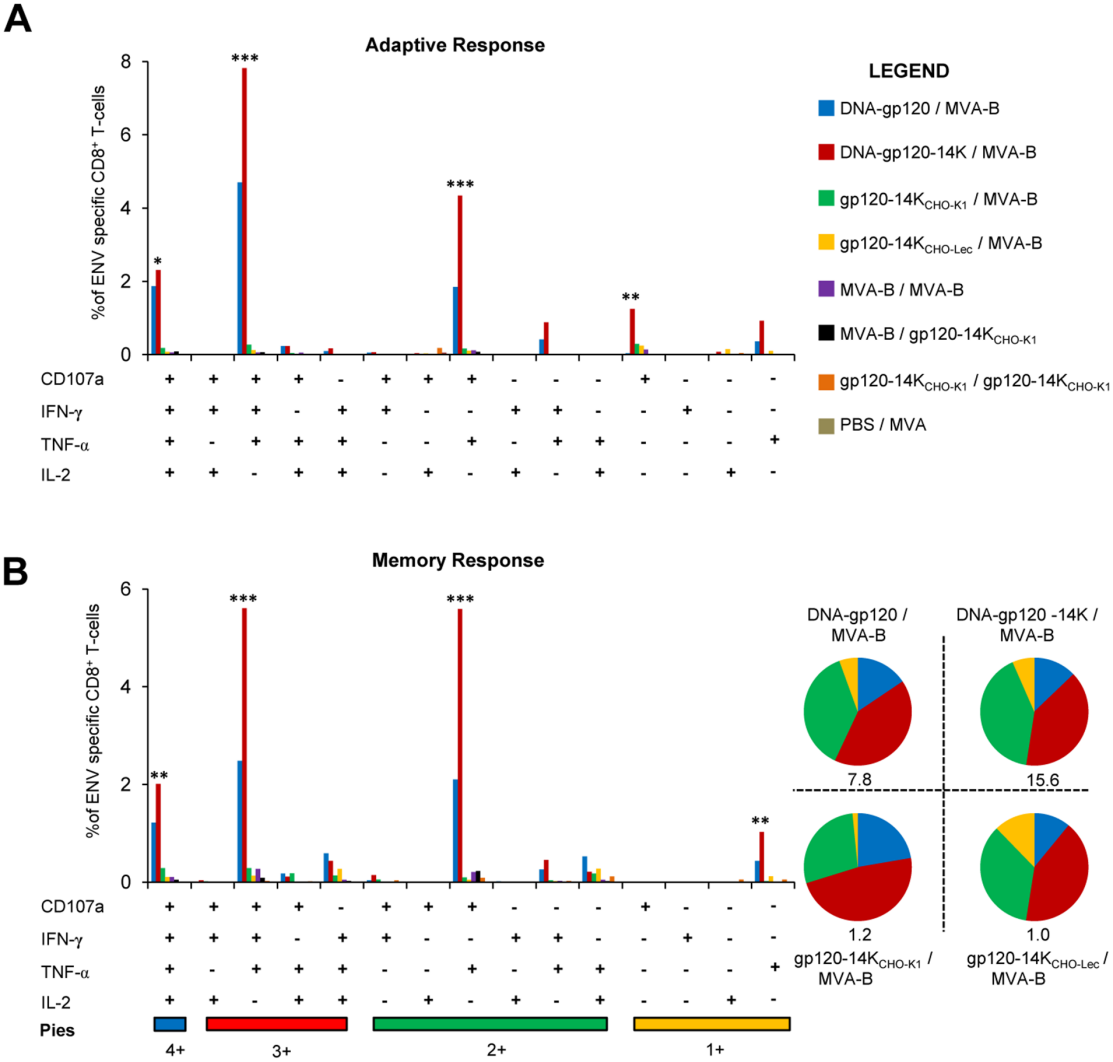
Enhancement of polyfunctional Env-specific CD8^+^ T-cells by HIV-1 gp120-14K. Env-specific CD8^+^ T-cell responses are based on the secretion of CD107a, IFN-γ, TNF-α and IL-2. Polyfunctionality is defined as the ability of the Env-specific CD8^+^ T-cells to secrete a combination of two or more cytokines. **(A)** Polyfunctionality of Env-specific CD8^+^ T-cells in the adaptive phase. **(B)** Polyfunctionality of Env-specific CD8^+^ T-cells in the memory phase. Pie charts represent the distribution of cells secreting different combination of cytokines and numbers below indicate the total magnitude. Data are representative of two independent experiments. Statistical significance between the PBS/MVA control animals and vaccinated animals within the polyfunctional population are represented. ** p < 0.005; *** p < 0.001.

In terms of the CD8^+^ T cells, DNA-gp120/MVA-B and DNA-gp120-14K/MVA-B immunization groups induced similar highly polyfunctional profile of Env-specific CD8^+^ T-cells, during the adaptive (Fig 7A) and memory (Fig 7B) phases. However, DNA-gp120-14K/MVA-B induced a significant increase in the magnitude of CD8^+^ T-cell populations producing CD107a^+^IFN-γ^+^TNF-α^+^IL-2^+^, CD107a^+^IFN-γ^+^TNF-α^+^, and CD107a^+^TNF-α^+^ in both the adaptive and memory phases (Fig 7A and 7B). Moreover, the gp120-14K_CHO-K1_/MVA-B immunization group also improved the polyfunctional CD8^+^ T-cell profile compared to all the other groups, with one third of the population being “quadruple positive” and more than half “triple positive”, albeit with lower magnitude (Fig 7B, pie charts). Additionally, polyfunctionality of Env-specific CD4^+^ and CD8^+^ T-cell memory immune responses induced by DNA-gp120/MVA-B and DNA-gp120-14K/MVA-B immunization groups was also observed when A20 cells transfected with DNA-gp120 were used as a stimulus instead of the Env peptides (S1C and S1D Fig).

Overall, DNA-gp120-14K priming/MVA-B boost was the best protocol, as it enhanced in immunized mice the quantity and quality of Env-specific CD4^+^ and CD8^+^ T-cell adaptive and memory immune responses; and it also provides a quantitatively higher immune response than protein/MVA protocol. This latter protocol triggered higher HIV-1-specific immune responses than MVA/protein, protein/protein or MVA/MVA immunizations.

### HIV-1 gp120-14K significantly improves the generation of long-term effector memory T-cells

**Fig 8:**
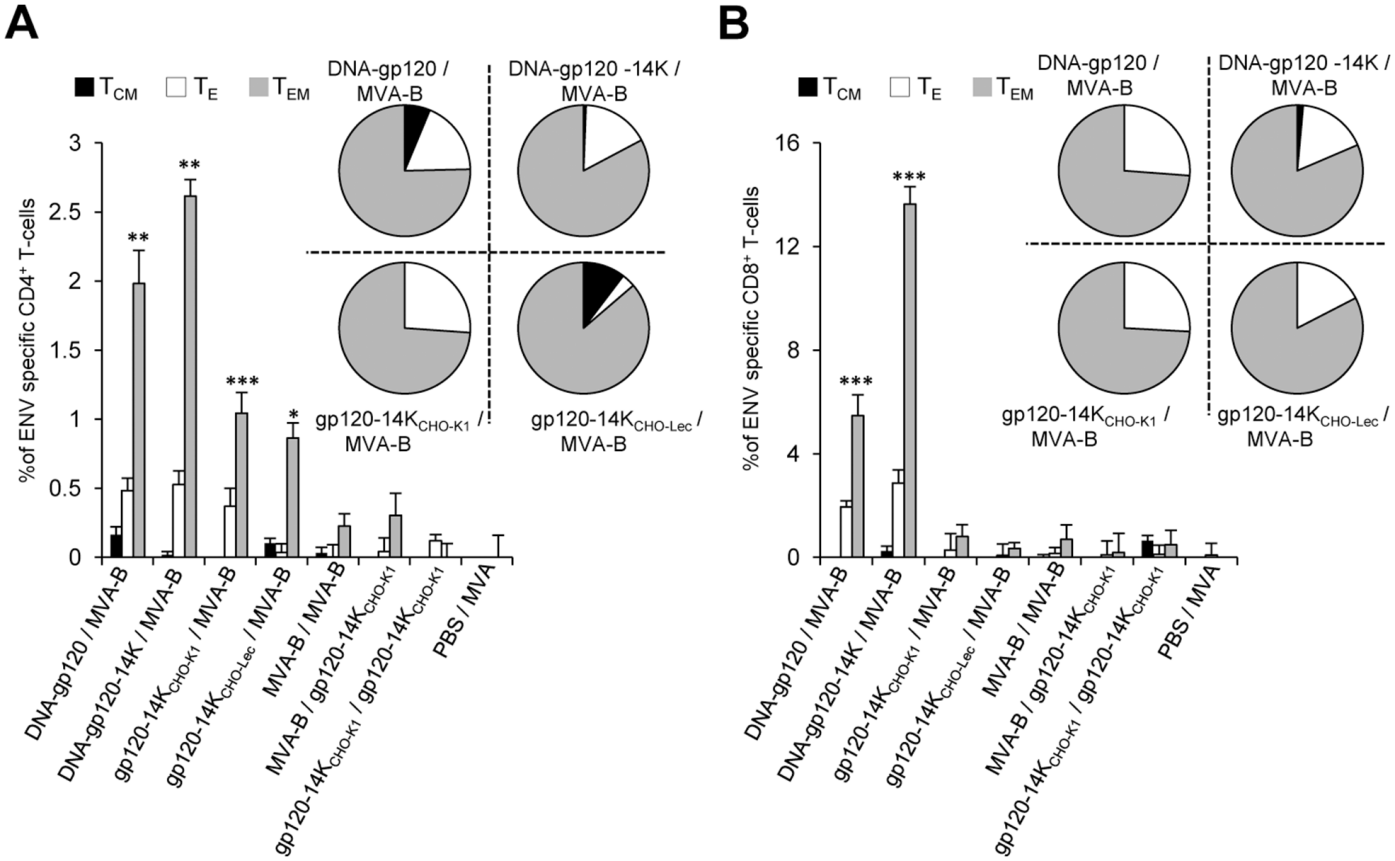
CD62L^low^CD127^high^ effector memory T-cells are enhanced by chimeric HIV-1 gp120-14K protein vaccine regimen. T-cells were differentiated based on CD62L and CD127 markers into central memory (T_CM_), effector memory (T_EM_) and effector T-cells (T_E_). The pie charts represent the distribution of different memory populations. **(A)** Total Env-specific CD4^+^ memory T-cell distribution and **(B)** Total Env-specific CD8^+^ memory T-cell distribution in the different vaccinated groups with its corresponding distribution shown in pie charts. Data are expressed as mean ± SD and are representative of two independent experiments. Statistical significances are shown between PBS/MVA control animals and the vaccinated animals. * p < 0.05; ** p < 0.005; *** p < 0.001.

We also determined the phenotype of the Env-specific memory CD4^+^ and CD8^+^ T-cells by measuring the expression of CD127 and CD62L surface markers, which allowed us to define the different memory subpopulations: central memory (T_CM_; CD127^+^/CD62L^+^), effector memory (T_EM_; CD127^+^/CD62L^-^), and effector (T_E_; CD127^-^/CD62L^-^) T cells [64, 65]. The results showed that most of the Env-specific memory CD4^+^ and CD8^+^ T-cells induced by vaccination with all the different immunization groups have a T_EM_ phenotype (Fig 8). Moreover, DNA-gp120-14K/MVA-B immunization group significantly improved the magnitude of T_EM_ CD4^+^ T-cells by 1.5 fold (*p* < 0.005) (Fig 8A) and T_EM_ CD8^+^ T-cells by 2.5 fold (*p* < 0.005) (Fig 8B), compared to DNA-gp120/MVA-B immunization group. Similar results were also observed when A20 cells transfected with gp120 were used as a stimulus instead of the Env peptides (S2A and S2B Fig).

These data indicate that the essential long-term effector memory T-cells, which known to be an important correlate of protection in the SIV infected macaque model [66], are significantly augmented by DNA-gp120-14K priming.

### Vaccination based on HIV-1 gp120-14K favors the induction of gp120-specific IgG2a and IgG3 over IgG1 antibodies

**Fig 9:**
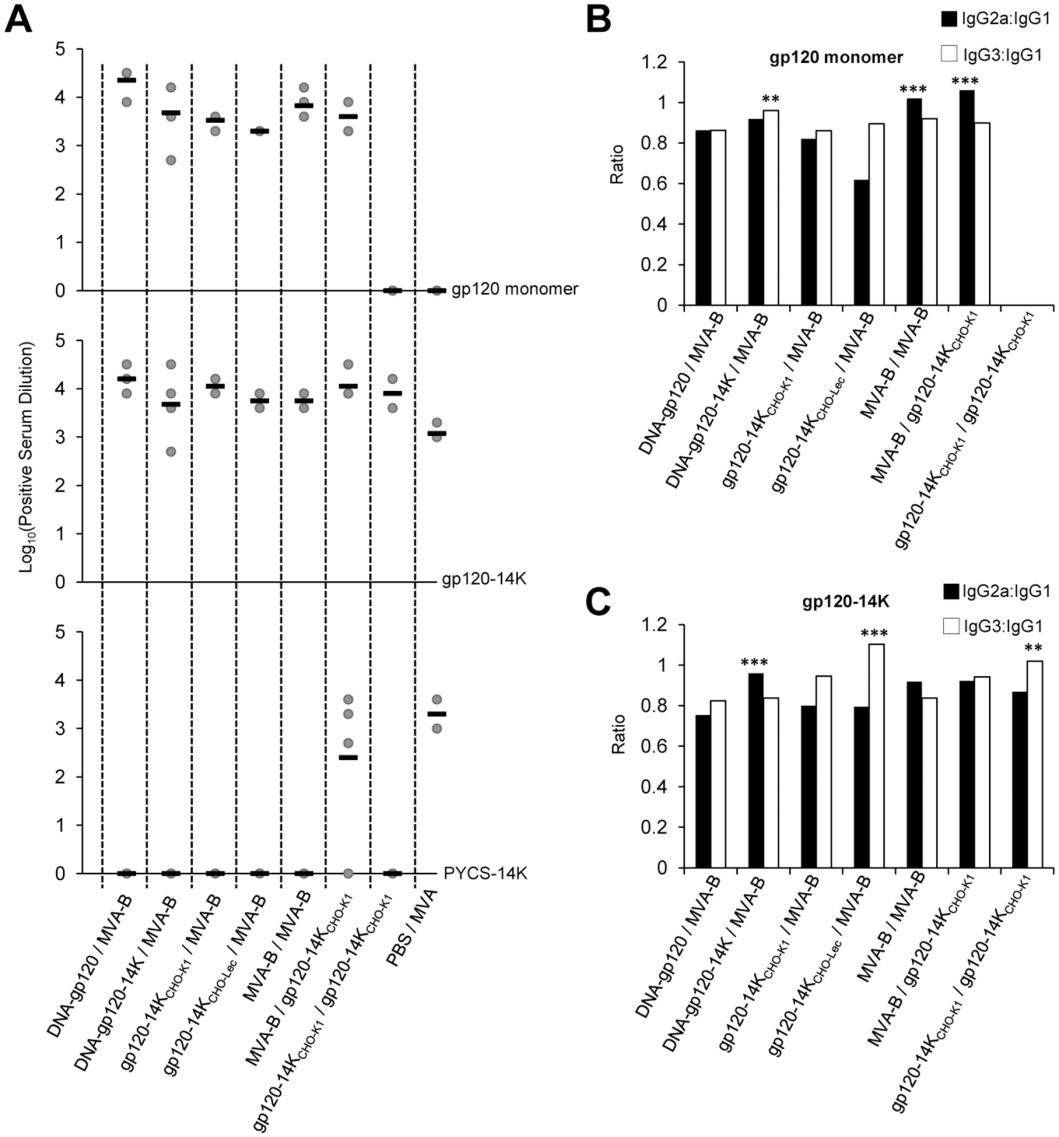
Vaccination based on HIV-1 gp120-14K skews humoral response towards a T_H_1 phenotype. **(A)** Serum of immunized mice from the memory phase were analyzed for total IgG response using ELISA plates coated with HIV-1 gp120-IIIB, HIV-1 gp120-14K_CHO-K1_ and PYCS-14K (an oligomeric fusion protein between circumsporozoite protein of malaria parasite *Plasmodium yoelii* and 14K). **(B and C)** Ratio of IgG2a or IgG3 antibodies to IgG1, measured against monomeric gp120 **(B)** and gp120-14K_CHO-K1_ protein **(C)**. The response was considered T_H_1 if the ratio between the average of IgG2a and IgG3 to IgG1 was greater than 1. Statistical significance between the DNA-gp120 primed animals to other groups was analyzed. ** p < 0.005; *** p < 0.001.

The RV144 phase III clinical trial correlated lower HIV infection risk with binding of antibodies to linear V2, V3 and V1/V2 epitopes, together with IgG3 antibodies against the Env V1/V2 (23–25). Therefore, we analyzed by ELISA in serum obtained at the memory phase from immunized mice, for the levels of gp120-specific total IgG, as well as for the different isotypes IgG1, IgG2a and IgG3 antibodies. The different immunizations were able to induce high titers of total IgG antibodies against the monomeric gp120 (except for the PBS/MVA control group), or against gp120-14K. A homologous prime/boost immunization with gp120-14K_CHO-K1_ protein produced antibodies which recognized epitopes exposed on the oligomeric gp120-14K protein, but not on the monomeric gp120 protein (Fig 9A). Comparative analysis of ratios of IgG2a and IgG3 over IgG1 revealed that these prime/boost protocols trigger a preferential induction of IgG2a and IgG3 isotype antibodies against gp120 and gp120-14K. Additionally, priming with gp120-14K_CHO-Lec_ protein and booster with MVA-B produced the higher ratios of IgG3 over IgG2a, against both gp120 (Fig 9B) and gp120-14K_CHO-K1_ (Fig 9C).

Therefore, HIV-1 gp120-14K is an effective immunogen as it induced good Env antibody responses with preferential IgG3 and IgG2a isotypes, thereby skewing the response towards a Th1 phenotype.

## Discussion

HIV/AIDS vaccine candidates in clinical trials based on Env protein have met with limited success since most of them were monomers and do not resemble the native trimeric form of the HIV-1 gp120 protein [67–69]. Even the RV144 phase III clinical trial, which showed modest protection against HIV-1 infection, comprised of a monomeric gp120 [11]. Thus, recent studies have clearly elucidated the importance of conformational integrity of HIV-1 gp120 protein in eliciting protective immune responses against HIV-1 [13, 46], and have emphasized the requirement of a trimeric HIV-1 gp120 protein to enhance the generation of neutralizing antibodies [46]. However, issues involving stability and glycosylation hampered the development of an effective trimeric HIV-1 gp120 protein. Thus far, generation of a more native-like HIV-1 gp140 was attained by mutating residues so as to stabilize the gp120/gp41 interaction or by the addition of a trimerization domain such as GCN4 [70, 71]. The generation of more stable trimers of gp140 has been recently obtained [46, 72, 73]. Therefore, while an array of native-like gp120 vaccine candidates have been developed there is interest in the development of novel Env vaccine candidates with enhanced immunogenicity at the B and T cell level. We hypothesized that the VACV 14K (A27) protein fused to HIV-1 gp120 should aid the oligomerization of gp120 leading to enhanced immunogenicity of the chimeric protein, as previously shown for the Plasmodium CS antigen with proven protective efficacy after its fusion to VACV 14K protein in the malaria murine model of infection [26]. Interestingly the coiled coil domain of 14K protein is similar to the heptad region 1 of HIV-1 gp41 [27, 74]. Our study here provides an in-depth analysis of a new oligomeric form of gp120 fused with the VACV 14K protein (gene A27L). We have produced purified oligomers of gp120-14K protein from CHO cells and defined specificity of binding to a panel of neutralizing monoclonal Abs, ability to activate innate responses in human moDCs and to trigger T and B cell immune responses in immunized mice.

Fusing VACV 14K protein to HIV-1 gp120 aided the formation of stable, soluble gp120-14K oligomers which were easily purified in high quantities from CHO cells under native conditions using lectin chromatography followed by SEC. The molecular weight of the gp120-14K based on SEC and BN-PAGE analysis corroborates with gp120 trimers [46, 70, 73]. Furthermore, apparent trimer-like proteins were also observed by electron microscopy. The fact that the gp120-14K protein remains oligomeric after SDS-PAGE denaturation under non-reducing conditions suggests that the protein is held together by inter monomer disulfide bonds. We generated two different HIV-1 gp120-14K proteins (gp120-14K_CHO-K1_ and gp120-14K_CHO-Lec_) differing in their glycosylation pattern so as to evaluate the effect of glycosylation on monoclonal Ab binding and immunogenicity. To remove complex glycans from gp120-14K, the protein was produced in CHO-Lec_3.2.8.1_ cells, which lack the enzyme GlcNAc transferase T_1_, required to process complex sugars [75]. Differential glycosylation of the protein did not obscure the binding of CD4 to the protein. However, we believe that the oligomeric assembly prevents the accessibility of Endo-H (β1–4 glycosidase) resulting in the strong binding of 2G12 antibody to gp120-14K_Deglycosylated_. On the other hand, binding of PG9 and PG16 antibodies to gp120-14K_Deglycosylated_ protein was abrogated but not with fully glycosylated gp120-14K_CHO-K1_ since these antibodies interact with Man_5_GlcNAc_2_-Asn_160_ [45], which lies very near to the glycan stem, and therefore could be easily accessed by the enzyme. Moreover, the gp120-14K protein reacted well with monoclonal Abs against a glycan-dependent epitope, N332 glycan V3 loop, the V3 loop and at the base of the V3 loop. These studies highlight the antigenic nature of gp120-14K.

Several studies have elucidated the role played by DCs in shaping immune responses against HIV-1 infection [76–78]. As an immunosuppressive agent, gp120 inhibits the activation of DCs, and is known to induce IL-10, promoting a Th2 response [79, 80]. In contrast, gp120-14K_CHO-K1_ was able to induce a Th1 response in human moDCs with a significant upregulation of IL-12, IFN-γ and TNF-α expression, cytokines which are known to aid DCs in priming T-cells [81]. This is consistent with our earlier report based on circumsporozoite-14K fusion protein of malaria where we showed that VACV 14K as such is not immunogenic and in fact the oligomerization of the protein is essential in driving the immune response [26]. Moreover, HIV-1 gp120-14K significantly enhanced RANTES production, an important β-chemokine that has been shown to be a potent suppressor of HIV-1 infection [82, 83], and in addition acts as a chemoattractant for monocytes and T-cells, an advantageous feature to enhance immunogenicity. However, the upregulation of IL-1β gene by gp120-14K was not reflected at protein level, indicating that the gp120-14K was able to enhance the production of pro-IL-1β but may require additional activation of the NLRP3 inflammasome complex to produce caspase-1 which cleaves pro-IL-1β into active IL-1β [84].

Previous studies have shown that gp120-based HIV/AIDS vaccine candidates generate IgG1 antibodies against gp120, promoting a Th2 humoral immune response. However, it is essential to induce a much broader antibody response against gp120, mediated by IgG2a and IgG3 antibodies which promote a Th1 humoral immune response [61, 85, 86]. This is reflected in our *in vivo* studies where vaccination based on gp120-14K priming (either as a DNA or as a protein) was able to induce higher levels of IgG2a and IgG3 antibodies than IgG1. In fact, IgG2a predominantly targets polysaccharides, while IgG3 have high affinity for F_C_ receptor which aids ADCC [87–89]. Therefore, higher levels of IgG2a and IgG3 antibodies targeting gp120-14K, associated with a homologous prime/boost based on gp120-14K_CHO-K1_ protein, is of prime importance. Recently, IgG3 antibodies directed against V_1_/V_2_ region of gp120 were identified as an important correlate of protection in vaccinated individuals [23]. Furthermore, the decrease in IgG2a antibodies in gp120-14K_CHO-Lec_ protein-primed immunized mice is due to the significant reduction in the complex sugars, since this antibody isotype has been reported to target mainly the carbohydrate moiety [88, 90]. Therefore, the ability of HIV-1 gp120-14K to skew the response in favor of a Th1 humoral immune response, especially IgG3, could prove beneficial in the generation of neutralizing antibodies.

Our results confirm that the robust Th1 innate immune responses primed by HIV-1 gp120-14K protein in moDCs were translated into strong adaptive and memory HIV-1-specific CD4^+^ and CD8^+^ T-cell immune responses. Previous reports have shown the association of increased CD4^+^ and CD8^+^ T-cell responses with reduced viremia [17, 62]. Moreover, a strong association of effector memory CD4^+^ Th1 cells producing IFN-γ^+^IL-2^+^ with reduced viremia in long-term nonprogressors (LTNPs) has been reported [91]. Another important correlate of protection in LTNPs, is the induction of polyfunctional CD8^+^ T-cells with 4 or more functions, especially associated with degranulation, IFN-γ and IL-2 [92]. Therefore, the significant increment in polyfunctionality of HIV-1-specific CD8^+^ T-cells by the DNA-gp120-14K/MVA-B immunization observed in this study is important for a vaccine immunogen. The high frequency of polyfunctional CD62L^-^CD127^+^ effector memory T-cells induced by DNA-gp120-14K/MVA-B prime-boost could help to provide long lasting immunity, the ultimate goal of any vaccine [65, 93, 94]. It should be noted that in this study we have not mixed the purified gp120-14K protein with adjuvants, as the VACV 14K protein provides an adjuvant effect. However, to further enhance the magnitude of HIV-1-specific B and T-cell immune responses by the purified gp120-14K other adjuvants currently in use in the HIV field could be considered.

In summary, these results demonstrate that the VACV 14K protein can help in the oligomerization and immunogenicity of HIV-1 Env when fused with gp120, generating an HIV-1 gp120-14K protein that can easily be purified from cultured cells, is recognized by a broad panel of HIV-1 neutralizing monoclonal antibodies and in immunized animals induces in prime/boost protocols, strong, broad, highly polyfunctional and memory gp120-specific T-cell responses, together with high titer of antibodies against gp120 of the class IgG2a and IgG3. This novel immunogen HIV-1 gp120-14K, delivered as DNA vector and/or purified protein, can be considered as a potential HIV vaccine candidate for broad T-cell and B-cell immune responses.

## Supporting Information

S1 Fig Characterization of the magnitude and polyfunctionality of Env-specific T-cell memory immune responses using gp120 transfected A20 cells as stimulus. A20 cells nucleofected with DNA-gp120 plasmid was used as a stimulus for evaluating T-cell memory immune responses against gp120. Vaccinated animals were sacrificed 2 months after boost and the splenocytes were stimulated with A20 cells nucleofected with gp120. The memory immune responses were analyzed as mentioned in Materials and Methods. **(A)** Total magnitude of Env-specific CD4^+^ T-cell responses. **(B)** Total magnitude of Env-specific CD8^+^ T-cell responses. **(C and D)** Polyfunctionality of Env-specific CD4^+^ **(C)** and CD8^+^ **(D)** T-cells in immunized animals. Pie charts represent the distribution of polyfunctional T cells. Data are representative of two independent experiments. * p < 0.05; ** p < 0.005; *** p < 0.001.
(TIF)

S2 Fig Characterization of the T-cell memory phenotype of Env-specific memory T-cells using gp120 transfected A20 cells as stimulus. A20 cells nucleofected with DNA-gp120 plasmid was used as a stimulus for evaluating the T-cell memory phenotype against gp120. Vaccinated animals were sacrificed 2 months after boost and the splenocytes were stimulated with A20 cells nucleofected with gp120. The memory immune responses were analyzed as mentioned in Materials and Methods. **(A)** Distribution of memory CD4^+^ T-cells. **(B)** Distribution of memory CD8^+^ T-cells. Pie charts represent the distribution of different population of memory T-cells. Statistical significances are shown between PBS/MVA control animals and the vaccinated animals. ** p < 0.005; *** p < 0.001.
(TIF)

## References

[1] Huang Y, Li Z, Xing H, Jiao Y, Ouyang Y, Liao L, et al Identification of the Critical Sites of NNRTI-Resistance in Reverse Transcriptase of HIV-1 CRF_BC Strains. PLoS One. 2014;9(4):e93804 Epub 2014/04/20. 10.1371/journal.pone.0093804 PONE-D-13-51575 [pii]. . 24743727 PMC3990534

[2] Pennings PS. HIV Drug Resistance: Problems and Perspectives. Infect Dis Rep. 2013;5(Suppl 1):e5 Epub 2014/01/29. 10.4081/idr.2013.s1.e5 24470969 PMC3892620

[3] McFarland EJ, Borkowsky W, Fenton T, Wara D, McNamara J, Samson P, et al Human immunodeficiency virus type 1 (HIV-1) gp120-specific antibodies in neonates receiving an HIV-1 recombinant gp120 vaccine. J Infect Dis. 2001;184(10):1331–5. Epub 2001/10/27. JID001160 [pii] 10.1086/323994 . 11679925

[4] Pantophlet R, Burton DR. GP120: target for neutralizing HIV-1 antibodies. Annu Rev Immunol. 2006;24:739–69. Epub 2006/03/23. 10.1146/annurev.immunol.24.021605.090557 . 16551265

[5] McCune JM, Rabin LB, Feinberg MB, Lieberman M, Kosek JC, Reyes GR, et al Endoproteolytic cleavage of gp160 is required for the activation of human immunodeficiency virus. Cell. 1988;53(1):55–67. Epub 1988/04/08. 0092-8674(88)90487-4 [pii]. . 2450679 10.1016/0092-8674(88)90487-4

[6] Deng H, Liu R, Ellmeier W, Choe S, Unutmaz D, Burkhart M, et al Identification of a major co-receptor for primary isolates of HIV-1. Nature. 1996;381(6584):661–6. Epub 1996/06/20. 10.1038/381661a0 . 8649511

[7] Dragic T. An overview of the determinants of CCR5 and CXCR4 co-receptor function. J Gen Virol. 2001;82(Pt 8):1807–14. Epub 2001/07/18. . 11457985 10.1099/0022-1317-82-8-1807

[8] Dalgleish AG, Beverley PC, Clapham PR, Crawford DH, Greaves MF, Weiss RA. The CD4 (T4) antigen is an essential component of the receptor for the AIDS retrovirus. Nature. 1984;312(5996):763–7. Epub 1984/12/20. . 6096719 10.1038/312763a0

[9] Piantadosi A, Panteleeff D, Blish CA, Baeten JM, Jaoko W, McClelland RS, et al Breadth of neutralizing antibody response to human immunodeficiency virus type 1 is affected by factors early in infection but does not influence disease progression. J Virol. 2009;83(19):10269–74. Epub 2009/07/31. JVI.01149-09 [pii] 10.1128/JVI.01149-09 19640996 PMC2748011

[10] Richman DD, Wrin T, Little SJ, Petropoulos CJ. Rapid evolution of the neutralizing antibody response to HIV type 1 infection. Proc Natl Acad Sci U S A. 2003;100(7):4144–9. Epub 2003/03/20. 10.1073/pnas.0630530100 0630530100 [pii]. 12644702 PMC153062

[11] Rerks-Ngarm S, Pitisuttithum P, Nitayaphan S, Kaewkungwal J, Chiu J, Paris R, et al Vaccination with ALVAC and AIDSVAX to prevent HIV-1 infection in Thailand. N Engl J Med. 2009;361(23):2209–20. Epub 2009/10/22. NEJMoa0908492 [pii] 10.1056/NEJMoa0908492 . 19843557

[12] Hoffenberg S, Powell R, Carpov A, Wagner D, Wilson A, Kosakovsky Pond S, et al Identification of an HIV-1 clade A envelope that exhibits broad antigenicity and neutralization sensitivity and elicits antibodies targeting three distinct epitopes. J Virol. 2013;87(10):5372–83. Epub 2013/03/08. JVI.02827-12 [pii] 10.1128/JVI.02827-12 23468492 PMC3648150

[13] Kovacs JM, Nkolola JP, Peng H, Cheung A, Perry J, Miller CA, et al HIV-1 envelope trimer elicits more potent neutralizing antibody responses than monomeric gp120. Proc Natl Acad Sci U S A. 2012;109(30):12111–6. Epub 2012/07/10. 1204533109 [pii] 10.1073/pnas.1204533109 22773820 PMC3409750

[14] Sundling C, Forsell MN, O'Dell S, Feng Y, Chakrabarti B, Rao SS, et al Soluble HIV-1 Env trimers in adjuvant elicit potent and diverse functional B cell responses in primates. J Exp Med. 2010;207(9):2003–17. Epub 2010/08/04. jem.20100025 [pii] 10.1084/jem.20100025 20679401 PMC2931166

[15] Julien JP, Lee JH, Cupo A, Murin CD, Derking R, Hoffenberg S, et al Asymmetric recognition of the HIV-1 trimer by broadly neutralizing antibody PG9. Proc Natl Acad Sci U S A. 2013;110(11):4351–6. Epub 2013/02/22. 1217537110 [pii] 10.1073/pnas.1217537110 23426631 PMC3600498

[16] Walker LM, Phogat SK, Chan-Hui PY, Wagner D, Phung P, Goss JL, et al Broad and potent neutralizing antibodies from an African donor reveal a new HIV-1 vaccine target. Science. 2009;326(5950):285–9. Epub 2009/09/05. 1178746 [pii] 10.1126/science.1178746 19729618 PMC3335270

[17] McMichael AJ, Koff WC. Vaccines that stimulate T cell immunity to HIV-1: the next step. Nat Immunol. 2014;15(4):319–22. Epub 2014/03/22. ni.2844 [pii] 10.1038/ni.2844 . 24646598 PMC4324504

[18] Streeck H, D'Souza MP, Littman DR, Crotty S. Harnessing CD4(+) T cell responses in HIV vaccine development. Nat Med. 2013;19(2):143–9. Epub 2013/02/08. nm.3054 [pii] 10.1038/nm.3054 23389614 PMC3626561

[19] Buckheit RW 3rd, Siliciano RF, Blankson JN. Primary CD8+ T cells from elite suppressors effectively eliminate non-productively HIV-1 infected resting and activated CD4+ T cells. Retrovirology. 2013;10:68 Epub 2013/07/03. 1742-4690-10-68 [pii] 10.1186/1742-4690-10-68 23816179 PMC3702406

[20] Rosenberg ES, Billingsley JM, Caliendo AM, Boswell SL, Sax PE, Kalams SA, et al Vigorous HIV-1-specific CD4+ T cell responses associated with control of viremia. Science. 1997;278(5342):1447–50. Epub 1997/12/31. . 9367954 10.1126/science.278.5342.1447

[21] Chung AW, Ghebremichael M, Robinson H, Brown E, Choi I, Lane S, et al Polyfunctional Fc-effector profiles mediated by IgG subclass selection distinguish RV144 and VAX003 vaccines. Sci Transl Med. 2014;6(228):228ra38 Epub 2014/03/22. 6/228/228ra38 [pii] 10.1126/scitranslmed.3007736 . 24648341

[22] Haynes BF, Gilbert PB, McElrath MJ, Zolla-Pazner S, Tomaras GD, Alam SM, et al Immune-correlates analysis of an HIV-1 vaccine efficacy trial. N Engl J Med. 2012;366(14):1275–86. Epub 2012/04/06. 10.1056/NEJMoa1113425 22475592 PMC3371689

[23] Yates NL, Liao HX, Fong Y, deCamp A, Vandergrift NA, Williams WT, et al Vaccine-induced Env V1-V2 IgG3 correlates with lower HIV-1 infection risk and declines soon after vaccination. Sci Transl Med. 2014;6(228):228ra39 Epub 2014/03/22. 6/228/228ra39 [pii] 10.1126/scitranslmed.3007730 . 24648342 PMC4116665

[24] Zolla-Pazner S, deCamp A, Gilbert PB, Williams C, Yates NL, Williams WT, et al Vaccine-induced IgG antibodies to V1V2 regions of multiple HIV-1 subtypes correlate with decreased risk of HIV-1 infection. PLoS One. 2014;9(2):e87572 Epub 2014/02/08. 10.1371/journal.pone.0087572 PONE-D-13-38754 [pii]. 24504509 PMC3913641

[25] Gottardo R, Bailer RT, Korber BT, Gnanakaran S, Phillips J, Shen X, et al Plasma IgG to linear epitopes in the V2 and V3 regions of HIV-1 gp120 correlate with a reduced risk of infection in the RV144 vaccine efficacy trial. PLoS One. 2013;8(9):e75665 Epub 2013/10/03. 10.1371/journal.pone.0075665 PONE-D-13-29733 [pii]. 24086607 PMC3784573

[26] Vijayan A, Gomez CE, Espinosa DA, Goodman AG, Sanchez-Sampedro L, Sorzano CO, et al Adjuvant-like effect of vaccinia virus 14K protein: a case study with malaria vaccine based on the circumsporozoite protein. J Immunol. 2012;188(12):6407–17. Epub 2012/05/23. jimmunol.1102492 [pii] 10.4049/jimmunol.1102492 . 22615208 PMC4181723

[27] Vazquez MI, Rivas G, Cregut D, Serrano L, Esteban M. The vaccinia virus 14-kilodalton (A27L) fusion protein forms a triple coiled-coil structure and interacts with the 21-kilodalton (A17L) virus membrane protein through a C-terminal alpha-helix. J Virol. 1998;72(12):10126–37. Epub 1998/11/13. 9811753 10.1128/jvi.72.12.10126-10137.1998 PMC110549

[28] Vazquez MI, Esteban M. Identification of functional domains in the 14-kilodalton envelope protein (A27L) of vaccinia virus. J Virol. 1999;73(11):9098–109. Epub 1999/10/09. 10516016 10.1128/jvi.73.11.9098-9109.1999 PMC112942

[29] Lai CF, Gong SC, Esteban M. Structural and functional properties of the 14-kDa envelope protein of vaccinia virus synthesized in Escherichia coli. J Biol Chem. 1990;265(36):22174–80. Epub 1990/12/25. . 2266120

[30] Chang TH, Chang SJ, Hsieh FL, Ko TP, Lin CT, Ho MR, et al Crystal structure of vaccinia viral A27 protein reveals a novel structure critical for its function and complex formation with A26 protein. PLoS Pathog. 2013;9(8):e1003563 Epub 2013/08/31. 10.1371/journal.ppat.1003563 PPATHOGENS-D-12-02948 [pii]. 23990784 PMC3749956

[31] Gomez CE, Najera JL, Jimenez EP, Jimenez V, Wagner R, Graf M, et al Head-to-head comparison on the immunogenicity of two HIV/AIDS vaccine candidates based on the attenuated poxvirus strains MVA and NYVAC co-expressing in a single locus the HIV-1BX08 gp120 and HIV-1(IIIB) Gag-Pol-Nef proteins of clade B. Vaccine. 2007;25(15):2863–85. Epub 2006/11/23. S0264-410X(06)01068-1 [pii] 10.1016/j.vaccine.2006.09.090 . 17113200

[32] Garcia-Arriaza J, Arnaez P, Gomez CE, Sorzano CO, Esteban M. Improving Adaptive and Memory Immune Responses of an HIV/AIDS Vaccine Candidate MVA-B by Deletion of Vaccinia Virus Genes (C6L and K7R) Blocking Interferon Signaling Pathways. PLoS One. 2013;8(6):e66894 Epub 2013/07/05. 10.1371/journal.pone.0066894 PONE-D-13-07187 [pii]. 23826170 PMC3694958

[33] Garcia-Arriaza J, Gomez CE, Sorzano CO, Esteban M. Deletion of the vaccinia virus N2L gene encoding an inhibitor of IRF3 improves the immunogenicity of modified vaccinia virus Ankara expressing HIV-1 antigens. J Virol. 2014;88(6):3392–410. Epub 2014/01/07. JVI.02723-13 [pii] 10.1128/JVI.02723-13 24390336 PMC3957918

[34] Garcia-Arriaza J, Najera JL, Gomez CE, Tewabe N, Sorzano CO, Calandra T, et al A candidate HIV/AIDS vaccine (MVA-B) lacking vaccinia virus gene C6L enhances memory HIV-1-specific T-cell responses. PLoS One. 2011;6(8):e24244 Epub 2011/09/13. 10.1371/journal.pone.0024244 PONE-D-11-06009 [pii]. 21909386 PMC3164197

[35] Santiago C, Celma ML, Stehle T, Casasnovas JM. Structure of the measles virus hemagglutinin bound to the CD46 receptor. Nat Struct Mol Biol. 2010;17(1):124–9. Epub 2009/12/17. nsmb.1726 [pii] 10.1038/nsmb.1726 . 20010840

[36] Garcia F, Bernaldo de Quiros JC, Gomez CE, Perdiguero B, Najera JL, Jimenez V, et al Safety and immunogenicity of a modified pox vector-based HIV/AIDS vaccine candidate expressing Env, Gag, Pol and Nef proteins of HIV-1 subtype B (MVA-B) in healthy HIV-1-uninfected volunteers: A phase I clinical trial (RISVAC02). Vaccine. 2011;29(46):8309–16. Epub 2011/09/13. S0264-410X(11)01368-5 [pii] 10.1016/j.vaccine.2011.08.098 . 21907749

[37] Garcia-Arriaza J, Najera JL, Gomez CE, Sorzano CO, Esteban M. Immunogenic profiling in mice of a HIV/AIDS vaccine candidate (MVA-B) expressing four HIV-1 antigens and potentiation by specific gene deletions. PLoS One. 2010;5(8):e12395 Epub 2010/09/03. 10.1371/journal.pone.0012395 20811493 PMC2927552

[38] Gomez CE, Najera JL, Perdiguero B, Garcia-Arriaza J, Sorzano CO, Jimenez V, et al The HIV/AIDS vaccine candidate MVA-B administered as a single immunogen in humans triggers robust, polyfunctional, and selective effector memory T cell responses to HIV-1 antigens. J Virol. 2011;85(21):11468–78. Epub 2011/08/26. JVI.05165-11 [pii] 10.1128/JVI.05165-11 21865377 PMC3194965

[39] Sambrook J, Russell DW. Calcium-phosphate-mediated Transfection of Eukaryotic Cells with Plasmid DNAs. CSH Protoc. 2006;2006(1). 10.1101/pdb.prot3871 . 22485343

[40] Scheres SH, Nunez-Ramirez R, Sorzano CO, Carazo JM, Marabini R. Image processing for electron microscopy single-particle analysis using XMIPP. Nat Protoc. 2008;3(6):977–90. Epub 2008/06/10. nprot.2008.62 [pii] 10.1038/nprot.2008.62 18536645 PMC2778070

[41] Sorzano CO, Bilbao-Castro JR, Shkolnisky Y, Alcorlo M, Melero R, Caffarena-Fernandez G, et al A clustering approach to multireference alignment of single-particle projections in electron microscopy. J Struct Biol. 2010;171(2):197–206. Epub 2010/04/07. S1047-8477(10)00088-2 [pii] 10.1016/j.jsb.2010.03.011 20362059 PMC2893300

[42] Delaloye J, Roger T, Steiner-Tardivel QG, Le Roy D, Knaup Reymond M, Akira S, et al Innate immune sensing of modified vaccinia virus Ankara (MVA) is mediated by TLR2-TLR6, MDA-5 and the NALP3 inflammasome. PLoS Pathog. 2009;5(6):e1000480 Epub 2009/06/23. 10.1371/journal.ppat.1000480 19543380 PMC2691956

[43] Garcia-Arriaza J, Cepeda V, Hallengard D, Sorzano CO, Kummerer BM, Liljestrom P, et al A novel poxvirus-based vaccine, MVA-CHIKV, is highly immunogenic and protects mice against chikungunya infection. J Virol. 2014;88(6):3527–47. Epub 2014/01/10. JVI.03418-13 [pii] 10.1128/JVI.03418-13 . 24403588 PMC3957920

[44] Najera JL, Gomez CE, Garcia-Arriaza J, Sorzano CO, Esteban M. Insertion of vaccinia virus C7L host range gene into NYVAC-B genome potentiates immune responses against HIV-1 antigens. PLoS One. 2010;5(6):e11406 Epub 2010/07/09. 10.1371/journal.pone.0011406 20613977 PMC2894869

[45] McLellan JS, Pancera M, Carrico C, Gorman J, Julien JP, Khayat R, et al Structure of HIV-1 gp120 V1/V2 domain with broadly neutralizing antibody PG9. Nature. 2011;480(7377):336–43. Epub 2011/11/25. nature10696 [pii] 10.1038/nature10696 22113616 PMC3406929

[46] Sanders RW, Derking R, Cupo A, Julien JP, Yasmeen A, de Val N, et al A next-generation cleaved, soluble HIV-1 Env Trimer, BG505 SOSIP.664 gp140, expresses multiple epitopes for broadly neutralizing but not non-neutralizing antibodies. PLoS Pathog. 2013;9(9):e1003618 Epub 2013/09/27. 10.1371/journal.ppat.1003618 PPATHOGENS-D-13-01512 [pii]. 24068931 PMC3777863

[47] Lynch RM, Tran L, Louder MK, Schmidt SD, Cohen M, Dersimonian R, et al The development of CD4 binding site antibodies during HIV-1 infection. J Virol. 2012;86(14):7588–95. Epub 2012/05/11. JVI.00734-12 [pii] 10.1128/JVI.00734-12 22573869 PMC3416294

[48] Watkins JD, Diaz-Rodriguez J, Siddappa NB, Corti D, Ruprecht RM. Efficiency of neutralizing antibodies targeting the CD4-binding site: influence of conformational masking by the V2 loop in R5-tropic clade C simian-human immunodeficiency virus. J Virol. 2011;85(23):12811–4. Epub 2011/10/01. JVI.05994-11 [pii] 10.1128/JVI.05994-11 21957314 PMC3209380

[49] Li Y, O'Dell S, Walker LM, Wu X, Guenaga J, Feng Y, et al Mechanism of neutralization by the broadly neutralizing HIV-1 monoclonal antibody VRC01. J Virol. 2011;85(17):8954–67. Epub 2011/07/01. JVI.00754-11 [pii] 10.1128/JVI.00754-11 21715490 PMC3165784

[50] Tran EE, Borgnia MJ, Kuybeda O, Schauder DM, Bartesaghi A, Frank GA, et al Structural mechanism of trimeric HIV-1 envelope glycoprotein activation. PLoS Pathog. 2012;8(7):e1002797 Epub 2012/07/19. 10.1371/journal.ppat.1002797 PPATHOGENS-D-12-01032 [pii]. 22807678 PMC3395603

[51] Moore PL, Gray ES, Wibmer CK, Bhiman JN, Nonyane M, Sheward DJ, et al Evolution of an HIV glycan-dependent broadly neutralizing antibody epitope through immune escape. Nat Med. 2012;18(11):1688–92. Epub 2012/10/23. nm.2985 [pii] 10.1038/nm.2985 23086475 PMC3494733

[52] Sanders RW, Venturi M, Schiffner L, Kalyanaraman R, Katinger H, Lloyd KO, et al The mannose-dependent epitope for neutralizing antibody 2G12 on human immunodeficiency virus type 1 glycoprotein gp120. J Virol. 2002;76(14):7293–305. Epub 2002/06/20. 12072528 10.1128/JVI.76.14.7293-7305.2002 PMC136300

[53] Zolla-Pazner S, Cohen SS, Krachmarov C, Wang S, Pinter A, Lu S. Focusing the immune response on the V3 loop, a neutralizing epitope of the HIV-1 gp120 envelope. Virology. 2008;372(2):233–46. 10.1016/j.virol.2007.09.024 . 18061228

[54] Walker LM, Huber M, Doores KJ, Falkowska E, Pejchal R, Julien JP, et al Broad neutralization coverage of HIV by multiple highly potent antibodies. Nature. 2011;477(7365):466–70. 10.1038/nature10373 21849977 PMC3393110

[55] Sok D, Laserson U, Laserson J, Liu Y, Vigneault F, Julien JP, et al The effects of somatic hypermutation on neutralization and binding in the PGT121 family of broadly neutralizing HIV antibodies. PLoS Pathog. 2013;9(11):e1003754 10.1371/journal.ppat.1003754 24278016 PMC3836729

[56] Pejchal R, Doores KJ, Walker LM, Khayat R, Huang PS, Wang SK, et al A potent and broad neutralizing antibody recognizes and penetrates the HIV glycan shield. Science. 2011;334(6059):1097–103. 10.1126/science.1213256 21998254 PMC3280215

[57] Julien JP, Sok D, Khayat R, Lee JH, Doores KJ, Walker LM, et al Broadly neutralizing antibody PGT121 allosterically modulates CD4 binding via recognition of the HIV-1 gp120 V3 base and multiple surrounding glycans. PLoS Pathog. 2013;9(5):e1003342 10.1371/journal.ppat.1003342 23658524 PMC3642082

[58] Gorny MK, Xu JY, Karwowska S, Buchbinder A, Zolla-Pazner S. Repertoire of neutralizing human monoclonal antibodies specific for the V3 domain of HIV-1 gp120. J Immunol. 1993;150(2):635–43. . 7678279

[59] Gorny MK, Xu JY, Gianakakos V, Karwowska S, Williams C, Sheppard HW, et al Production of site-selected neutralizing human monoclonal antibodies against the third variable domain of the human immunodeficiency virus type 1 envelope glycoprotein. Proc Natl Acad Sci U S A. 1991;88(8):3238–42. 2014246 10.1073/pnas.88.8.3238 PMC51421

[60] Shingai M, Nishimura Y, Klein F, Mouquet H, Donau OK, Plishka R, et al Antibody-mediated immunotherapy of macaques chronically infected with SHIV suppresses viraemia. Nature. 2013;503(7475):277–80. 10.1038/nature12746 24172896 PMC4133787

[61] Bielinska AU, Janczak KW, Landers JJ, Markovitz DM, Montefiori DC, Baker JR Jr. Nasal immunization with a recombinant HIV gp120 and nanoemulsion adjuvant produces Th1 polarized responses and neutralizing antibodies to primary HIV type 1 isolates. AIDS Res Hum Retroviruses. 2008;24(2):271–81. Epub 2008/02/12. 10.1089/aid.2007.0148 . 18260780

[62] Freel SA, Saunders KO, Tomaras GD. CD8(+)T-cell-mediated control of HIV-1 and SIV infection. Immunol Res. 2011;49(1–3):135–46. Epub 2010/12/21. 10.1007/s12026-010-8177-7 . 21170741 PMC8259511

[63] Wherry EJ, Ahmed R. Memory CD8 T-cell differentiation during viral infection. J Virol. 2004;78(11):5535–45. Epub 2004/05/14. 10.1128/JVI.78.11.5535-5545.2004 78/11/5535 [pii]. 15140950 PMC415833

[64] Huster KM, Busch V, Schiemann M, Linkemann K, Kerksiek KM, Wagner H, et al Selective expression of IL-7 receptor on memory T cells identifies early CD40L-dependent generation of distinct CD8+ memory T cell subsets. Proc Natl Acad Sci U S A. 2004;101(15):5610–5. Epub 2004/03/27. 10.1073/pnas.0308054101 0308054101 [pii]. 15044705 PMC397444

[65] Sallusto F, Geginat J, Lanzavecchia A. Central memory and effector memory T cell subsets: function, generation, and maintenance. Annu Rev Immunol. 2004;22:745–63. Epub 2004/03/23. 10.1146/annurev.immunol.22.012703.104702 . 15032595

[66] Hansen SG, Ford JC, Lewis MS, Ventura AB, Hughes CM, Coyne-Johnson L, et al Profound early control of highly pathogenic SIV by an effector memory T-cell vaccine. Nature. 2011;473(7348):523–7. 10.1038/nature10003 21562493 PMC3102768

[67] Gilbert PB, Peterson ML, Follmann D, Hudgens MG, Francis DP, Gurwith M, et al Correlation between immunologic responses to a recombinant glycoprotein 120 vaccine and incidence of HIV-1 infection in a phase 3 HIV-1 preventive vaccine trial. J Infect Dis. 2005;191(5):666–77. Epub 2005/02/03. JID33334 [pii] 10.1086/428405 . 15688279

[68] Jones NG, DeCamp A, Gilbert P, Peterson ML, Gurwith M, Cao H. AIDSVAX immunization induces HIV-specific CD8+ T-cell responses in high-risk, HIV-negative volunteers who subsequently acquire HIV infection. Vaccine. 2009;27(7):1136–40. Epub 2008/12/17. S0264-410X(08)01582-X [pii] 10.1016/j.vaccine.2008.11.071 19071176 PMC2676722

[69] Pitisuttithum P, Gilbert P, Gurwith M, Heyward W, Martin M, van Griensven F, et al Randomized, double-blind, placebo-controlled efficacy trial of a bivalent recombinant glycoprotein 120 HIV-1 vaccine among injection drug users in Bangkok, Thailand. J Infect Dis. 2006;194(12):1661–71. Epub 2006/11/17. JID36424 [pii] 10.1086/508748 . 17109337

[70] Sanders RW, Vesanen M, Schuelke N, Master A, Schiffner L, Kalyanaraman R, et al Stabilization of the soluble, cleaved, trimeric form of the envelope glycoprotein complex of human immunodeficiency virus type 1. J Virol. 2002;76(17):8875–89. Epub 2002/08/07. 12163607 10.1128/JVI.76.17.8875-8889.2002 PMC136973

[71] Yang X, Farzan M, Wyatt R, Sodroski J. Characterization of stable, soluble trimers containing complete ectodomains of human immunodeficiency virus type 1 envelope glycoproteins. J Virol. 2000;74(12):5716–25. Epub 2000/05/24. 10823881 10.1128/jvi.74.12.5716-5725.2000 PMC112061

[72] Pugach P, Ozorowski G, Cupo A, Ringe R, Yasmeen A, de Val N, et al A native-like SOSIP.664 trimer based on an HIV-1 subtype B env gene. J Virol. 2015;89(6):3380–95. 10.1128/JVI.03473-14 25589637 PMC4337520

[73] Harris A, Borgnia MJ, Shi D, Bartesaghi A, He H, Pejchal R, et al Trimeric HIV-1 glycoprotein gp140 immunogens and native HIV-1 envelope glycoproteins display the same closed and open quaternary molecular architectures. Proc Natl Acad Sci U S A. 2011;108(28):11440–5. Epub 2011/06/29. 1101414108 [pii] 10.1073/pnas.1101414108 21709254 PMC3136299

[74] Chan DC, Fass D, Berger JM, Kim PS. Core structure of gp41 from the HIV envelope glycoprotein. Cell. 1997;89(2):263–73. Epub 1997/04/18. S0092-8674(00)80205-6 [pii]. . 9108481 10.1016/s0092-8674(00)80205-6

[75] Chen W, Stanley P. Five Lec1 CHO cell mutants have distinct Mgat1 gene mutations that encode truncated N-acetylglucosaminyltransferase I. Glycobiology. 2003;13(1):43–50. Epub 2003/03/14. 10.1093/glycob/cwg003 cwg003 [pii]. . 12634323

[76] Fantuzzi L, Purificato C, Donato K, Belardelli F, Gessani S. Human immunodeficiency virus type 1 gp120 induces abnormal maturation and functional alterations of dendritic cells: a novel mechanism for AIDS pathogenesis. J Virol. 2004;78(18):9763–72. Epub 2004/08/28. 10.1128/JVI.78.18.9763-9772.2004 78/18/9763 [pii]. 15331709 PMC515003

[77] Luban J. Innate immune sensing of HIV-1 by dendritic cells. Cell Host Microbe. 2012;12(4):408–18. Epub 2012/10/23. S1931-3128(12)00318-6 [pii] 10.1016/j.chom.2012.10.002 23084911 PMC3619430

[78] Sandgren KJ, Smed-Sorensen A, Forsell MN, Soldemo M, Adams WC, Liang F, et al Human plasmacytoid dendritic cells efficiently capture HIV-1 envelope glycoproteins via CD4 for antigen presentation. J Immunol. 2013;191(1):60–9. Epub 2013/06/05. jimmunol.1202489 [pii] 10.4049/jimmunol.1202489 . 23729440 PMC4471340

[79] Daly LM, Johnson PA, Donnelly G, Nicolson C, Robertson J, Mills KH. Innate IL-10 promotes the induction of Th2 responses with plasmid DNA expressing HIV gp120. Vaccine. 2005;23(7):963–74. Epub 2004/12/18. S0264-410X(04)00697-8 [pii] 10.1016/j.vaccine.2004.03.072 . 15603899

[80] Shan M, Klasse PJ, Banerjee K, Dey AK, Iyer SP, Dionisio R, et al HIV-1 gp120 mannoses induce immunosuppressive responses from dendritic cells. PLoS Pathog. 2007;3(11):e169 Epub 2007/11/07. 07-PLPA-RA-0438 [pii] 10.1371/journal.ppat.0030169 17983270 PMC2048530

[81] Paganin C, Frank I, Trinchieri G. Priming for high interferon-gamma production induced by interleukin-12 in both CD4+ and CD8+ T cell clones from HIV-infected patients. J Clin Invest. 1995;96(3):1677–82. Epub 1995/09/01. 10.1172/JCI118209 7657839 PMC185796

[82] Crawford A, Angelosanto JM, Nadwodny KL, Blackburn SD, Wherry EJ. A role for the chemokine RANTES in regulating CD8 T cell responses during chronic viral infection. PLoS Pathog. 2011;7(7):e1002098 Epub 2011/08/05. 10.1371/journal.ppat.1002098 PPATHOGENS-D-10-00148 [pii]. 21814510 PMC3141034

[83] Scarlatti G, Tresoldi E, Bjorndal A, Fredriksson R, Colognesi C, Deng HK, et al In vivo evolution of HIV-1 co-receptor usage and sensitivity to chemokine-mediated suppression. Nat Med. 1997;3(11):1259–65. Epub 1997/11/14. . 9359702 10.1038/nm1197-1259

[84] Church LD, Cook GP, McDermott MF. Primer: inflammasomes and interleukin 1beta in inflammatory disorders. Nat Clin Pract Rheumatol. 2008;4(1):34–42. Epub 2008/01/04. ncprheum0681 [pii] 10.1038/ncprheum0681 . 18172447

[85] Rao M, Matyas GR, Vancott TC, Birx DL, Alving CR. Immunostimulatory CpG motifs induce CTL responses to HIV type I oligomeric gp140 envelope protein. Immunol Cell Biol. 2004;82(5):523–30. Epub 2004/10/14. ICB1283 [pii] 10.1111/j.0818-9641.2004.01283.x . 15479438

[86] Visciano ML, Tuen M, Gorny MK, Hioe CE. In vivo alteration of humoral responses to HIV-1 envelope glycoprotein gp120 by antibodies to the CD4-binding site of gp120. Virology. 2008;372(2):409–20. Epub 2007/12/07. S0042-6822(07)00734-9 [pii] 10.1016/j.virol.2007.10.044 18054978 PMC2288784

[87] Jefferis R, Pound J, Lund J, Goodall M. Effector mechanisms activated by human IgG subclass antibodies: clinical and molecular aspects. Review article. Ann Biol Clin (Paris). 1994;52(1):57–65. Epub 1994/01/01. . 8210076

[88] Madassery JV, Kwon OH, Lee SY, Nahm MH. IgG2 subclass deficiency: IgG subclass assays and IgG2 concentrations among 8015 blood donors. Clin Chem. 1988;34(7):1407–13. Epub 1988/07/01. . 3292083

[89] Tomaras GD, Haynes BF. HIV-1-specific antibody responses during acute and chronic HIV-1 infection. Curr Opin HIV AIDS. 2009;4(5):373–9. Epub 2010/01/06. 01222929-200909000-00006 [pii]. 20048700 10.1097/COH.0b013e32832f00c0 PMC3133462

[90] Visciano ML, Tagliamonte M, Tornesello ML, Buonaguro FM, Buonaguro L. Effects of adjuvants on IgG subclasses elicited by virus-like particles. J Transl Med. 2012;10:4 Epub 2012/01/10. 1479-5876-10-4 [pii] 10.1186/1479-5876-10-4 22221900 PMC3311067

[91] Potter SJ, Lacabaratz C, Lambotte O, Perez-Patrigeon S, Vingert B, Sinet M, et al Preserved central memory and activated effector memory CD4+ T-cell subsets in human immunodeficiency virus controllers: an ANRS EP36 study. J Virol. 2007;81(24):13904–15. Epub 2007/10/12. JVI.01401-07 [pii] 10.1128/JVI.01401-07 17928341 PMC2168869

[92] Makedonas G, Betts MR. Living in a house of cards: re-evaluating CD8+ T-cell immune correlates against HIV. Immunol Rev. 2011;239(1):109–24. Epub 2011/01/05. 10.1111/j.1600-065X.2010.00968.x 21198668 PMC3025661

[93] Fonseca SG, Procopio FA, Goulet JP, Yassine-Diab B, Ancuta P, Sekaly RP. Unique features of memory T cells in HIV elite controllers: a systems biology perspective. Curr Opin HIV AIDS. 2011;6(3):188–96. Epub 2011/03/25. . 21430529 10.1097/COH.0b013e32834589a1

[94] Kaech SM, Wherry EJ, Ahmed R. Effector and memory T-cell differentiation: implications for vaccine development. Nat Rev Immunol. 2002;2(4):251–62. Epub 2002/05/11. 10.1038/nri778 . 12001996

