# Supplementary figures and images for: A Chimeric HIV-1 gp120 Fused with Vaccinia Virus 14K (A27) Protein as an HIV Immunogen
Source: PLoS One. 2015 Jul 24;10(7):e0133595. doi: 10.1371/journal.pone.0133595 (PMC4514760; doi:10.1371/journal.pone.0133595)

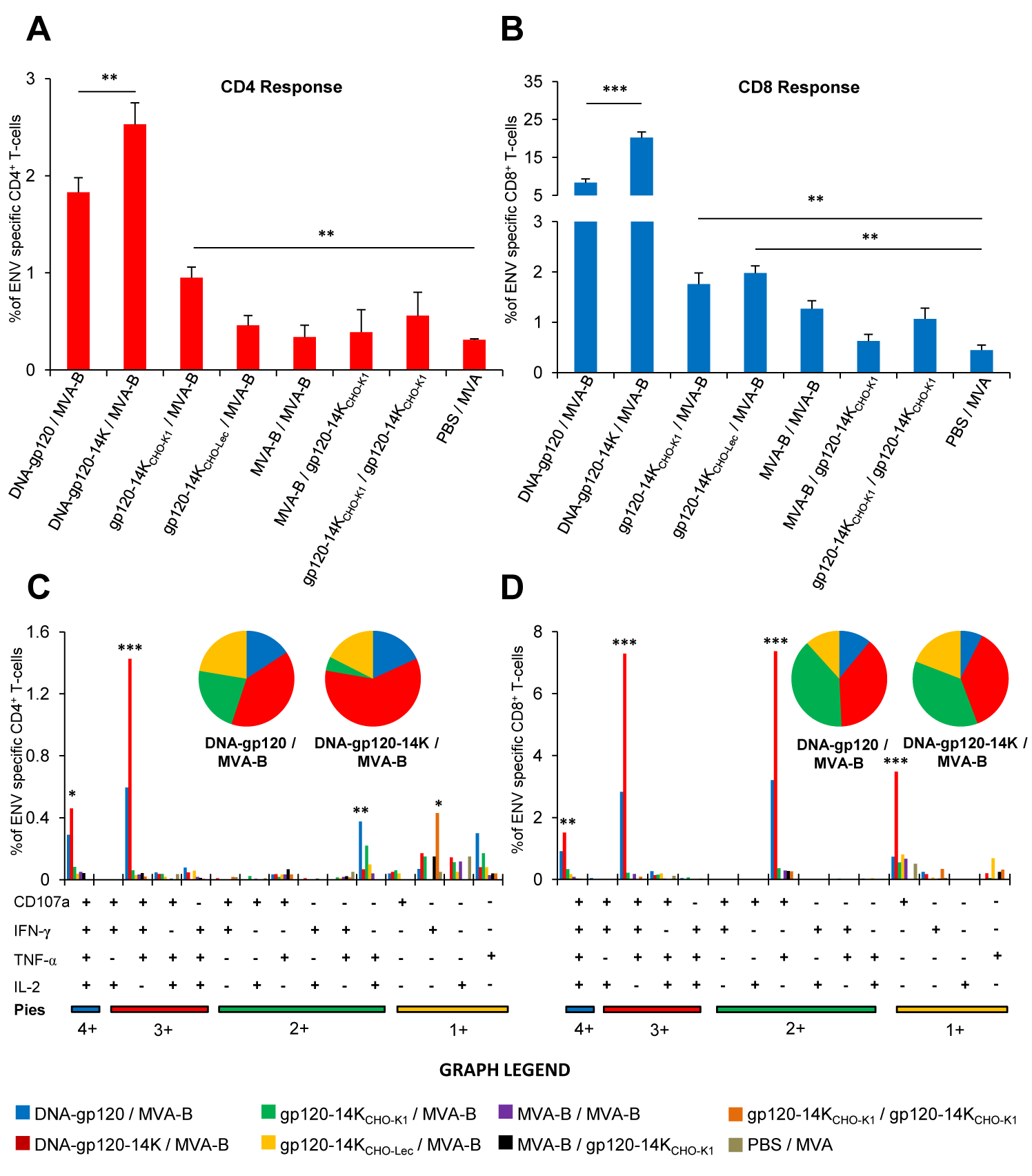

Supplement: S1 Fig — A20 cells nucleofected with DNA-gp120 plasmid was used as a stimulus for evaluating T-cell memory immune responses against gp120. Vaccinated animals were sacrificed 2 months after boost and the splenocytes were stimulated with A20 cells nucleofected with gp120. The memory immune responses were analyzed as mentioned in Materials and Methods. (A) Total magnitude of Env-specific CD4+ T-cell responses. (B) Total magnitude of Env-specific CD8+ T-cell responses. (C and D) Polyfunctionality of Env-specific CD4+ (C) and CD8+ (D) T-cells in immunized animals. Pie charts represent the distribution of polyfunctional T cells. Data are representative of two independent experiments. * p < 0.05; ** p < 0.005; *** p < 0.001. (TIF) [file pone.0133595.s001.tif]

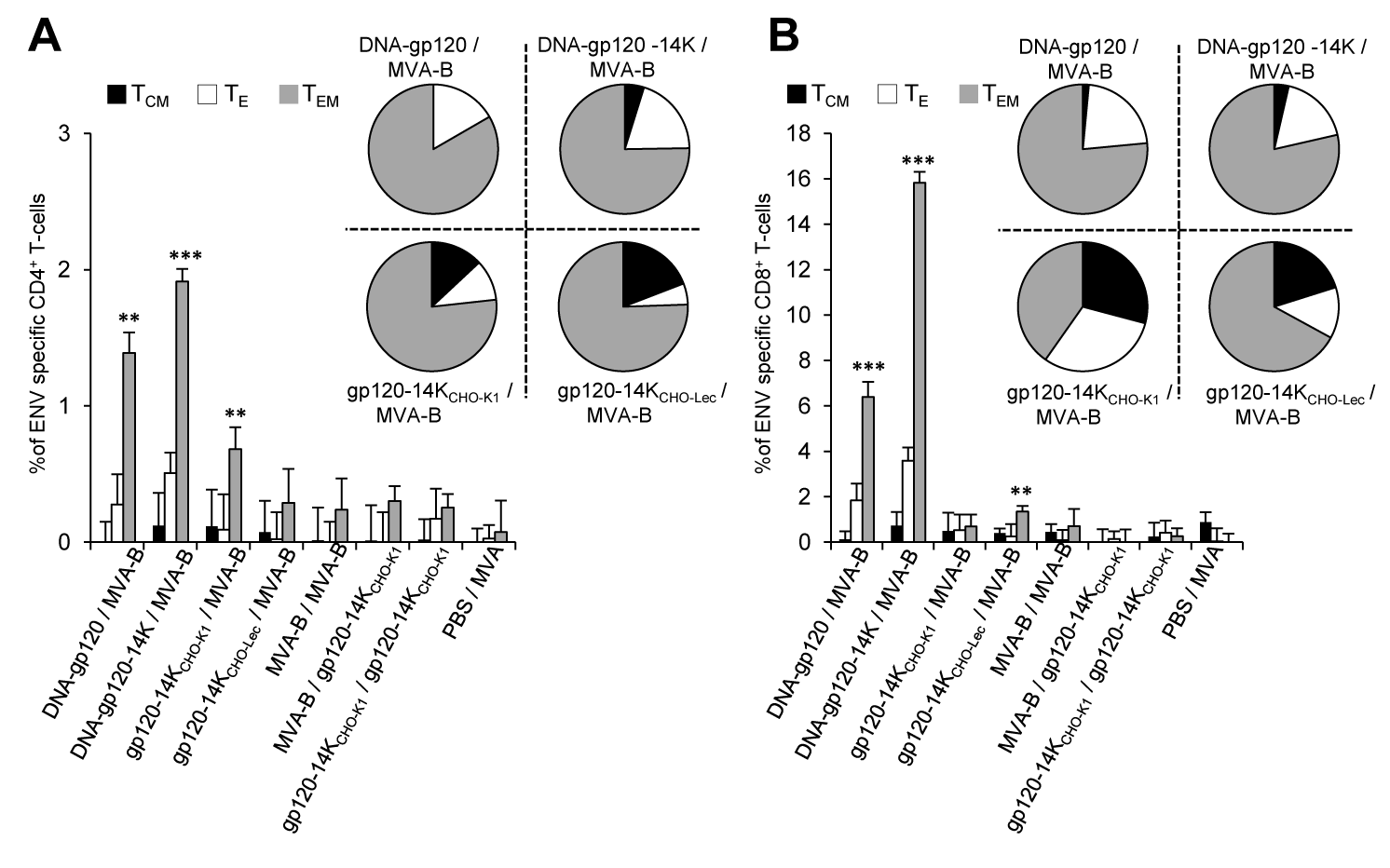

Supplement: S2 Fig — A20 cells nucleofected with DNA-gp120 plasmid was used as a stimulus for evaluating the T-cell memory phenotype against gp120. Vaccinated animals were sacrificed 2 months after boost and the splenocytes were stimulated with A20 cells nucleofected with gp120. The memory immune responses were analyzed as mentioned in Materials and Methods. (A) Distribution of memory CD4+ T-cells. (B) Distribution of memory CD8+ T-cells. Pie charts represent the distribution of different population of memory T-cells. Statistical significances are shown between PBS/MVA control animals and the vaccinated animals. ** p < 0.005; *** p < 0.001. (TIF) [file pone.0133595.s002.tif]
